# Cytosolic PSD-95 interactor alters functional organization of neural circuits and AMPA receptor signaling independent of PSD-95 binding

**DOI:** 10.1162/netn_a_00173

**Published:** 2021-02-01

**Authors:** Ana R. Rodriguez, Erin D. Anderson, Kate M. O’Neill, Przemyslaw P. McEwan, Nicholas F. Vigilante, Munjin Kwon, Barbara F. Akum, Tamara M. Stawicki, David F. Meaney, Bonnie L. Firestein

**Affiliations:** Department of Cell Biology and Neuroscience, Rutgers, The State University of New Jersey, Piscataway, NJ, USA; Biomedical Engineering Graduate Program, Rutgers, The State University of New Jersey, Piscataway, NJ, USA; Department of Bioengineering, University of Pennsylvania, Philadelphia, PA, USA; Department of Cell Biology and Neuroscience, Rutgers, The State University of New Jersey, Piscataway, NJ, USA; Biomedical Engineering Graduate Program, Rutgers, The State University of New Jersey, Piscataway, NJ, USA; Institute for Physical Science and Technology, University of Maryland, College Park, MD, USA; Department of Cell Biology and Neuroscience, Rutgers, The State University of New Jersey, Piscataway, NJ, USA; Graduate Program in Cellular and Molecular Pharmacology, Rutgers University, New Brunswick, NJ, USA; Department of Bioengineering, University of Pennsylvania, Philadelphia, PA, USA; Department of Cell Biology and Neuroscience, Rutgers, The State University of New Jersey, Piscataway, NJ, USA; Department of Cell Biology and Neuroscience, Rutgers, The State University of New Jersey, Piscataway, NJ, USA; Department of Cell Biology and Neuroscience, Rutgers, The State University of New Jersey, Piscataway, NJ, USA; Department of Bioengineering, University of Pennsylvania, Philadelphia, PA, USA; Department of Neurosurgery, University of Pennsylvania, Philadelphia, PA, USA; Department of Cell Biology and Neuroscience, Rutgers, The State University of New Jersey, Piscataway, NJ, USA

**Keywords:** Cypin, AMPA receptors, Microelectrode array, Electrophysiology, Hippocampal neurons, Neural circuits

## Abstract

Cytosolic PSD-95 interactor (cypin) regulates many aspects of neuronal development and function, ranging from dendritogenesis to synaptic protein localization. While it is known that removal of postsynaptic density protein-95 (PSD-95) from the postsynaptic density decreases synaptic N-methyl-D-aspartate (NMDA) receptors and that cypin overexpression protects neurons from NMDA-induced toxicity, little is known about cypin’s role in AMPA receptor clustering and function. Experimental work shows that cypin overexpression decreases PSD-95 levels in synaptosomes and the PSD, decreases PSD-95 clusters/μm^2^, and increases mEPSC frequency. Analysis of microelectrode array (MEA) data demonstrates that cypin or cypinΔPDZ overexpression increases sensitivity to CNQX (cyanquixaline) and AMPA receptor-mediated decreases in spike waveform properties. Network-level analysis of MEA data reveals that cypinΔPDZ overexpression causes networks to be resilient to CNQX-induced changes in local efficiency. Incorporating these findings into a computational model of a neural circuit demonstrates a role for AMPA receptors in cypin-promoted changes to networks and shows that cypin increases firing rate while changing network functional organization, suggesting cypin overexpression facilitates information relay but modifies how information is encoded among brain regions. Our data show that cypin promotes changes to AMPA receptor signaling independent of PSD-95 binding, shaping neural circuits and output to regions beyond the hippocampus.

## Author Summary

We used lentivirus to overexpress cytosolic PSD-95 interactor (cypin) and a cypin mutant that cannot bind PSD-95 (cypinΔPDZ) to understand how cypin regulates synaptic signaling. Using biochemical and electrophysiological approaches, we show that cypin, but not the cypin mutant, regulates synaptic PSD-95 content. Surprisingly, cypin overexpression increases AMPA receptor function independent of its role in PSD-95 localization, while cypinΔPDZ overexpression causes networks to be resistant to CNQX-mediated changes to local efficiency. We then developed a computational model to account for the effects of cypin overexpression, finding that our simulated model also demonstrates a role for AMPA receptors in cypin-promoted changes to neural circuits. These results support a role for cypin in controlling synaptic function at synapses that is distinct from regulation of PSD-95 function.

## INTRODUCTION

Neurons transfer information to each other via signaling complexes assembled at synapses. Defects in synaptogenesis and changes to synapse number result in neurocognitive disorders, including schizophrenia and autism, and in neurodegenerative disorders, such as Alzheimer’s disease and dementia (Kulkarni & Firestein, 2012). Postsynaptic density protein-95 (PSD-95), a member of the membrane-associated guanylate kinase family, is an essential scaffolding protein, and it plays an important role in assembling signaling complexes at excitatory synapses (Brenman et al., 1996; Cho et al., 1992; Kim et al., 1998; Kistner et al., 1993). PSD-95 contains three PDZ domains, which are protein-protein interaction motifs (Kim & Sheng, 2004) and which bind to multiple receptors and ion channels and their accessory proteins (Cohen et al., 1996; Kim et al., 1995; Kornau et al., 1995) that contain the carboxyl terminal PDZ-binding consensus sequence Thr/Ser-X-Val/Ile-COOH (Kornau et al., 1995; Sheng & Wyszynski, 1997). These PDZ domains serve to aggregate neurotransmitter receptors, such as the NMDA and α-amino-3-hydroxy-5-methyl-4-isoxazolepropionic acid (AMPA) receptors that are activated by the excitatory neurotransmitter glutamate, and their downstream enzymes, such as neuronal nitric oxide synthase (NOS1, nNOS), thus promoting subcellular protein compartmentalization and ensuring selective activation of signal transduction cascades at synaptic sites (Scott & Zuker, 1997, 1998). PSD-95 also promotes spine formation and maturation (El-Husseini et al., 2000; Okabe et al., 1999; Prange & Murphy, 2001). Thus, the regulation of PSD-95 expression/degradation, clustering, and localization may play an important role in establishment and maintenance of proper synaptic connections during development and throughout adulthood (Matus, 2000).

Cypin (cytosolic PSD-95 interactor) contains a four-residue PDZ-binding motif at its carboxyl terminus and binds to PSD-95 at PDZ 1 and 2 (Firestein et al., 1999). The binding of cypin to PSD-95 results in decreased synaptic localization of PSD-95 and family members, such as synapse-associated protein-102 (SAP-102; Firestein et al., 1999). When cypin is overexpressed with exogenous fluorescently tagged PSD-95 or SAP-102, the number of synaptic clusters of these proteins significantly decreases (Firestein et al., 1999). Furthermore, overexpression of a cypin mutant that lacks the PDZ-binding motif does not influence the clustering of PSD-95 or SAP-102.

The postsynaptic density undergoes structural remodeling during synaptic maturation due to a number of molecular changes, including the addition of new proteins (Steward & Schuman, 2001) and protein turnover (Marrs et al., 2001; Okabe et al., 1999) at the synapse. Dynamically regulated protein degradation occurs at the postsynaptic density, and this process can alter synaptic receptors and signaling to downstream effectors (Ehlers, 2003). In fact, PSD-95 is ubiquitinated and rapidly disappears from synaptic sites when the NMDA receptor is stimulated (Colledge et al., 2003). In addition, PSD-95 levels decrease with AMPA-stimulated GluR endocytosis, which itself is regulated by the ubiquitin-proteasome system (Bingol & Schuman, 2004). Thus, by decreasing synaptic PSD-95 localization, cypin may play a role in modifying glutamate receptor signaling at the synapse.

Here we use biochemical, electrophysiological, and computational techniques to assess whether cypin influences AMPA receptor signaling and how this influence may affect neuronal information processing. We first show that overexpression of cypin decreases the levels of PSD-95 in synaptosomes and at the PSD and also decreases the number of PSD-95 clusters in cultured hippocampal neurons. Knockdown of cypin does not change the levels of PSD-95 at synapses but does increase the number of PSD-95 clusters. Consistent with our previous results, overexpression of cypin results in increased miniature excitatory postsynaptic current (mEPSC) frequency but not amplitude. Overexpression of a cypin mutant lacking the PDZ-binding motif (cypinΔPDZ) that does not bind PSD-95 (Firestein et al., 1999) also increases mEPSC frequency; however, in contrast to cypin overexpression, cypinΔPDZ overexpression results in increased PSD-95 at the PSD but does not affect the number of PSD-95 clusters. Microelectrode array (MEA) analysis demonstrates that overexpression of either cypin or cypinΔPDZ results in sensitivity to cyanquixaline (6-cyano-7-nitroquinoxaline-2,3-dione; CNQX), a competitive AMPA/kainate receptor antagonist, and in AMPA receptor-mediated changes to spike rate, burstlet rate, Fano factor, interspike interval, and coefficient of variation. Moreover, network-level analysis of MEA data reveals that overexpression of cypinΔPDZ, but not cypin, makes networks more resilient to CNQX-induced changes in local efficiency. Using a computational model of a neuronal network, we mimicked these experimental findings by adjusting AMPA receptor conductance, connection density, and presynaptic activity. Our simulations suggest that cypin overexpression facilitates information relay but may modify how information is encoded. Taken together, our results suggest cypin plays an important role in changing both synaptic- and network-scale characteristics of neural circuits.

## RESULTS

### Cypin Alters the Subcellular Distribution of PSD-95

**Figure 1.**
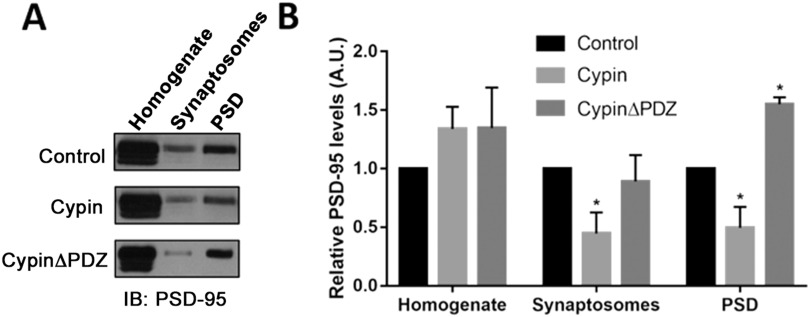
Cypin overexpression alters the subcellular distribution of endogenous PSD-95. (A) Representative blots showing decreased levels of PSD-95 in synaptosomes and postsynaptic density (PSD) of cultured hippocampal neurons overexpressing cypin as compared with levels in control cultures that express EGFP. (B) Densitometric analysis of PSD-95 protein relative to the control condition shows decreased PSD-95 in synaptosomes and PSD after cypin overexpression (*p < 0.05 as determined by two-way ANOVA followed by Dunnett’s multiple comparison test; *n* = 5 independent experiments for control, *n* = 5 for cypin overexpression, and *n* = 3 for cypinΔPDZ overexpression).

We previously reported that overexpression of cypin decreases the number of clusters of exogenously expressed PSD-95 (Firestein et al., 1999); however, the effects of altering cypin expression on endogenous PSD-95 are not known. To address this, we performed synaptosomal fractionation of lysates from cultured hippocampal neurons that overexpress cypin (Chen et al., 2005; Firestein et al., 1999) from day in vitro (DIV) 10–21. We isolated synaptosomes, composed of the presynaptic terminal, the postsynaptic membrane, and the postsynaptic density, and we also purified the postsynaptic density (Bai & Witzmann, 2007). We found that when cypin was overexpressed, there was a significant decrease in PSD-95 present in synaptosomes and postsynaptic density (PSD) fractions, where PSD-95 is typically enriched (Pak et al., 2001), versus PSD-95 levels in control neurons expressing enhanced green fluorescent protein (GFP; Figure 1). Furthermore, when cypinΔPDZ, which does not bind PSD-95, was overexpressed, we did not observe changes to PSD-95 present in synaptosomes; however, overexpression of this mutant resulted in increased PSD-95 at the postsynaptic density. Our findings are in agreement with our previous report (Firestein et al., 1999), which demonstrated that cypin decreases synaptic clustering of PSD-95 and that the presence of the PDZ-binding motif of cypin is necessary for this effect.

**Figure 2.**
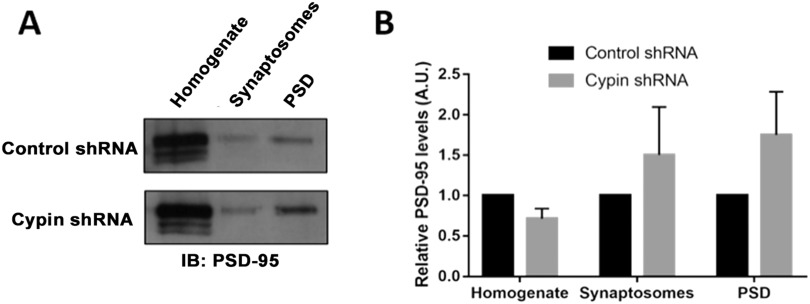
Cypin knockdown does not affect the subcellular distribution of PSD-95. (A) Representative blots and (B) densitometric analysis of PSD-95 protein levels in subcellular fractions after control shRNA or cypin knockdown. No statistical significance was found as calculated by two-way ANOVA followed by Sidak’s multiple comparison test (*n* = 6 independent experiments for both control shRNA and cypin shRNA conditions).

We further investigated the effect of altering cypin levels on PSD-95 localization and performed synaptosomal fractionation after cypin knockdown. Interestingly, we did not observe changes in PSD-95 localization after cypin knockdown when compared with control knockdown (Figure 2). Since we only achieved a partial knockdown with this method (i.e., 40% as we reported in Chen & Firestein, 2007), it is possible that the endogenous cypin levels that remain are sufficient to allow for correct PSD-95 localization.

**Figure 3.**
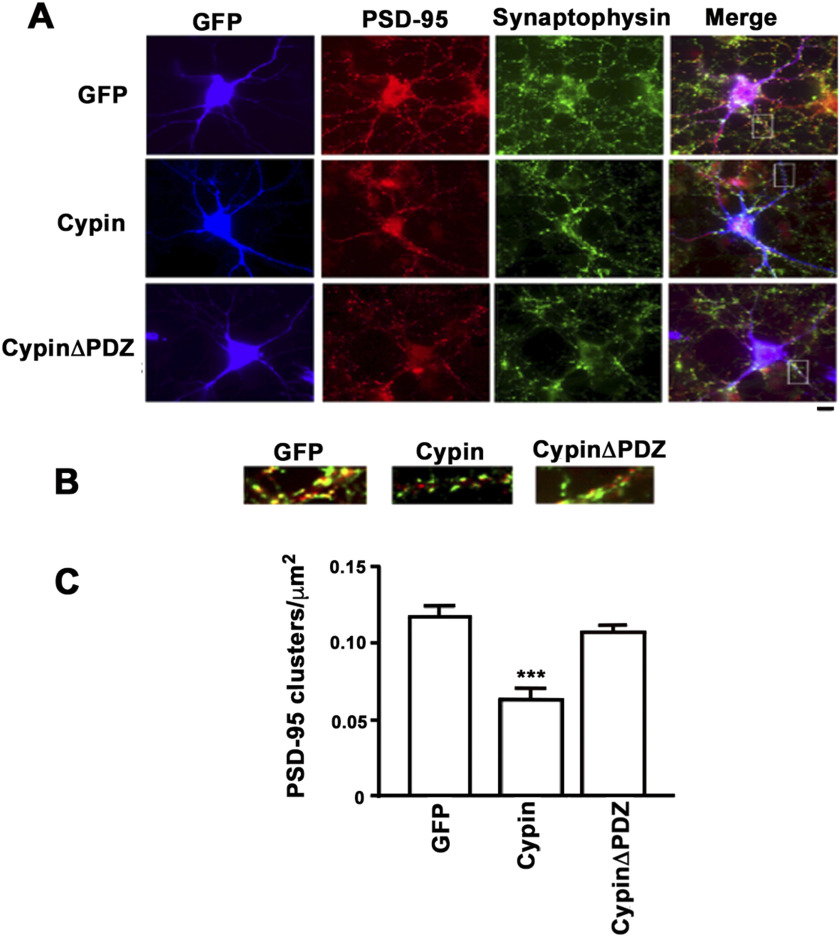
Cypin regulates PSD-95 localization. (A) Hippocampal neurons were transfected with cDNAs encoding the indicated constructs on DIV10 (blue) and immunostained for PSD-95 (red) and synaptophysin (green). Scale bar = 10 μm. (B) Magnification of areas indicated in panel A. (C) The number of PSD-95 family clusters per area was assessed on DIV17 (*n* = 20 for GFP, *n* = 22 for cypin, and *n* = 16 for cypinΔPDZ; ***p < 0.001 as determined by ANOVA followed by Dunnett’s multiple comparisons test as compared with GFP control).

**Figure 4.**
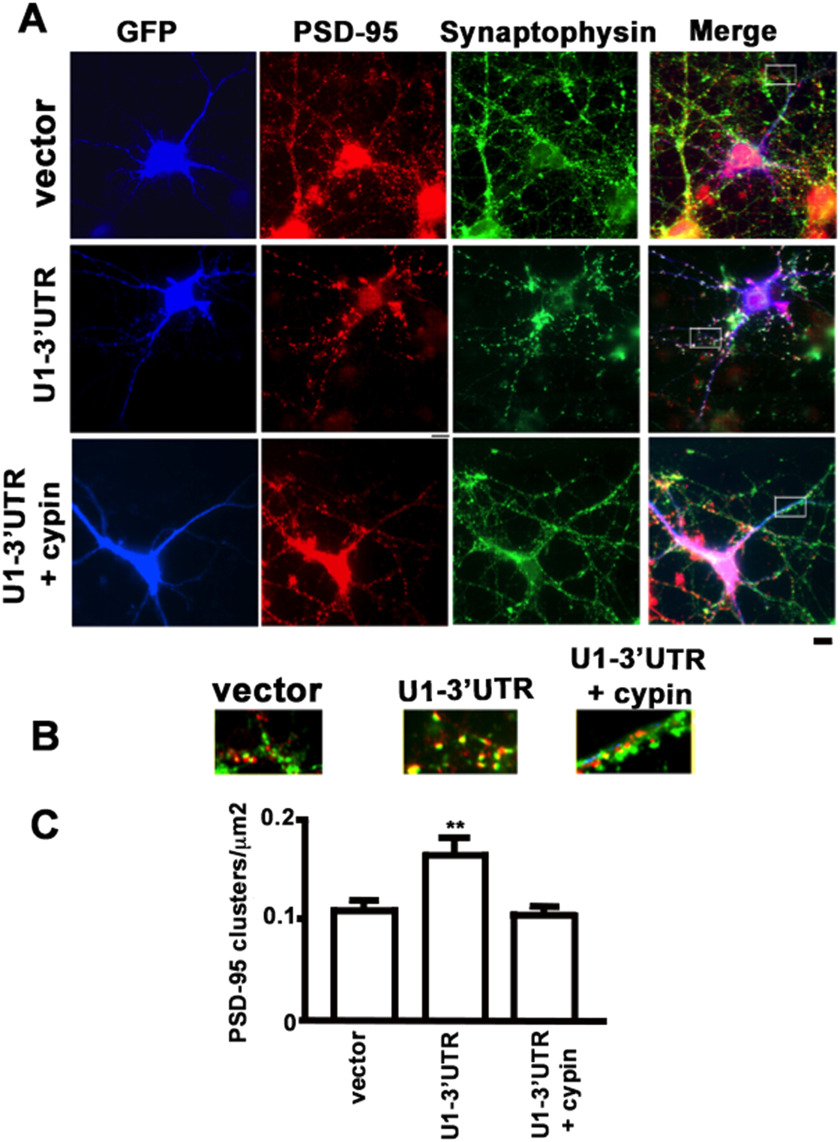
Knockdown of cypin protein expression increases PSD-95 clustering. Expression of U1-3′UTR results in attenuation of cypin protein expression (Akum et al., 2004). U1-3′UTR + cypin is constructed in a plasmid with bicistronic coding for GFP-cypin lacking the 3′UTR (U1 recognition site) and U1-3′UTR. (A) Hippocampal neurons were transfected with cDNAs encoding the indicated constructs on DIV10 (blue) and immunostained for PSD-95 (red) and synaptophysin (green). Scale bar = 10 μm. (B) Magnification of areas indicated in panel A. GFP-Cypin restores PSD-95 clusters to control values (vector). (C) Quantitation of clusters/area. Results shown are for DIV17, although results were similar for DIV12 (*n* = 14 for vector (control), *n* = 20 for U1-3′UTR, and *n* = 16 for U1-3′UTR + cypin. **p < 0.01 as determined by ANOVA followed by Dunnett’s multiple comparisons test as compared with vector control).

Synaptosomal fractionation provides information about the total amount of PSD-95 at the synapse; however, it does not give a measure of changes to PSD-95 clusters, that is, synapses containing PSD-95. Thus, we overexpressed GFP, cypin, or cypinΔPDZ in cultured hippocampal neurons and immunostained for endogenous PSD-95 and the presynaptic marker synaptophysin, which apposes postsynaptic components. When hippocampal neurons were transfected with cDNAs encoding cypin, the number of synaptic PSD-95 clusters per unit area was significantly reduced (Figure 3). Similarly, neurons transfected with cDNAs encoding 5′-end mutated U1 snRNAs that knock down cypin protein levels (Akum et al., 2004) demonstrate larger PSD-95 clusters, and coexpression of a U1 snRNA-resistant cypin construct restores PSD-95 cluster numbers to control levels (Figure 4). Taken together with the data in Figure 2, our studies suggest that cypin plays a role in PSD-95 localization, specifically by regulating the number of synaptic sites that contain PSD-95.

### Cypin Overexpression Increases Synaptic Transmission

**Figure 5.**
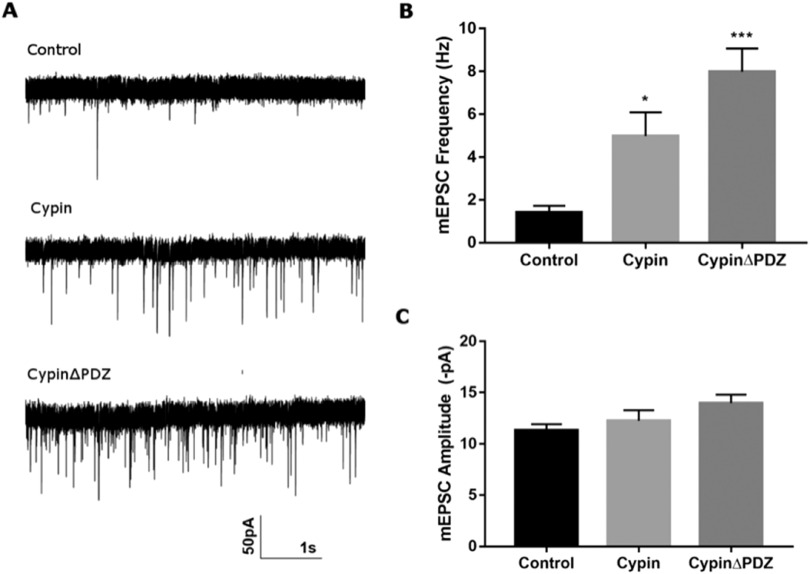
Overexpression of cypin or cypinΔPDZ results in increased frequency of mEPSCs. (A) Representative traces of mEPSCs. (B) Overexpression of cypin and cypinΔPDZ increases the frequency of mEPSCs. (C) The amplitude of mEPSCs remains unchanged after overexpression (*p < 0.05 and ***p < 0.001 as determined by one-way ANOVA followed by Tukey’s multiple comparisons test; *n* = 12 for control, *n* = 15 for cypin, and *n* = 15 for cypinΔPDZ).

To determine whether cypin regulates synaptic transmission, we performed whole-cell patch-clamp recordings of mEPSCs in hippocampal neurons. We transduced neurons with lentivirus to either overexpress or knock down cypin on DIV14 and recorded mEPSCs at DIV21. Overexpression of cypin or cypinΔPDZ results in an increase in the frequency of mEPSCs while their amplitude remains unchanged (Figure 5). Cypin knockdown results in no change to mEPSC frequency but increases amplitude (Swiatkowski et al., 2018). Taken together, these data suggest that the effect of cypin overexpression on the frequency of mEPSCs is independent of cypin binding to PSD-95 or PSD-95 family members.

### Networks Overexpressing Cypin Exhibit Increased AMPA Receptor Function

Changes to PSD-95 expression and localization influence the synaptic targeting and trafficking of glutamate receptors, resulting in alterations to the electrical activity of glutamatergic synapses (Beique et al., 2006; El-Husseini et al., 2000; Keith & El-Husseini, 2008; Yudowski et al., 2013). PSD-95 indirectly interacts with AMPAR subunits through stargazin and influences the efficiency of AMPAR trafficking (Bredt & Nicoll, 2003; Vandenberghe et al., 2005). To investigate whether cypin-promoted changes to PSD-95 protein levels and synaptic function affect AMPAR-mediated synaptic transmission, we cultured hippocampal neurons on MEAs and performed a baseline recording on DIV10. Immediately after this recording, we transduced MEA cultures with GFP, cypin, or cypinΔPDZ and monitored expression until DIV14 when the next recording was performed with increasing amounts of the AMPAR antagonist CNQX.

**Figure 6.**
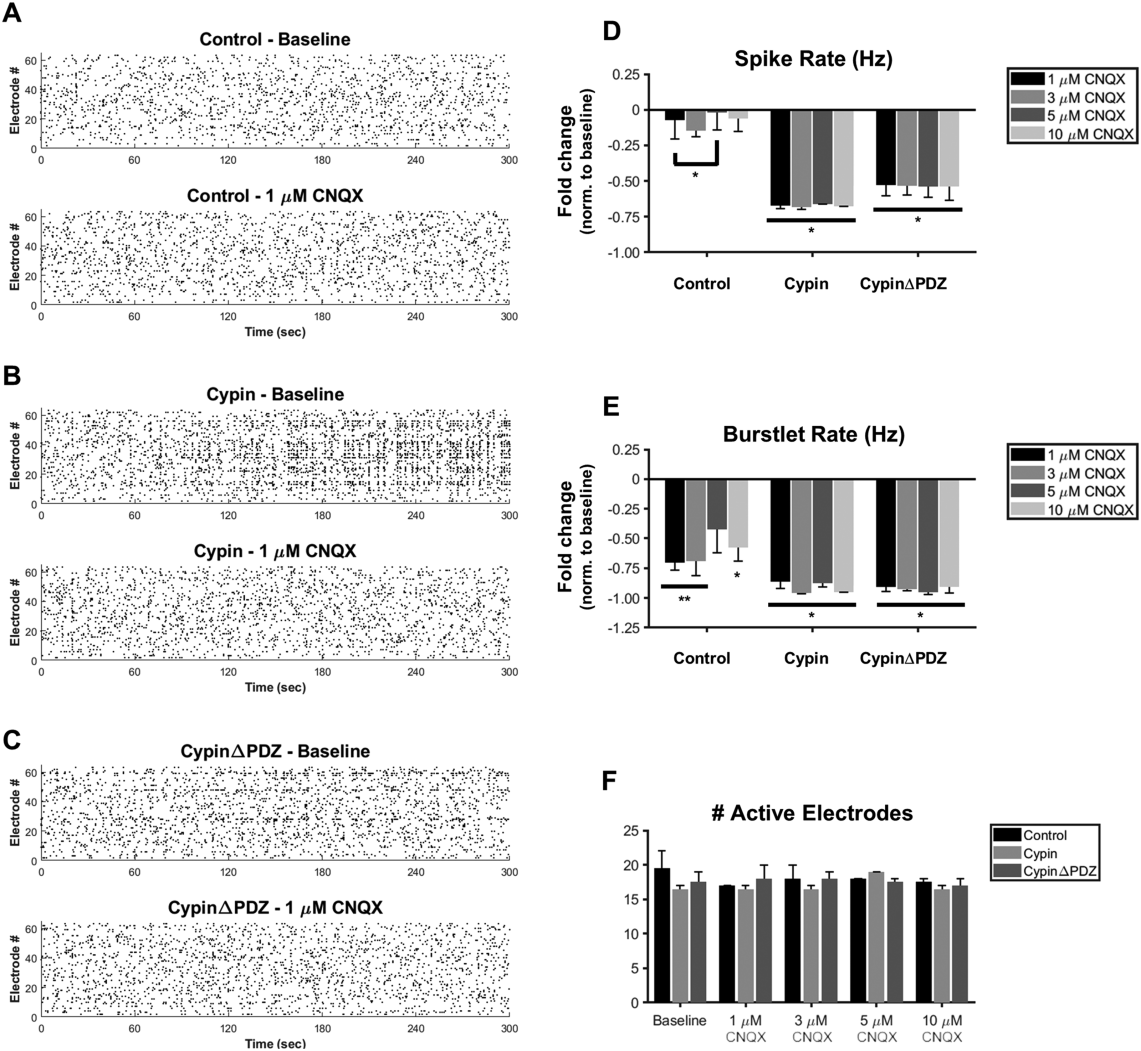
Networks of neurons overexpressing cypin exhibit higher sensitivity to CNQX. (A–C) Representative raster plots showing baseline activity (top) and activity after treatment with CNQX (bottom) for (A) control networks, (B) networks overexpressing cypin, and (C) networks overexpressing cypinΔPDZ. We chose 1 μM CNQX as the representative treatment condition because spiking and bursting activity were generally not significantly different between any of the CNQX treatment conditions (significant differences found only when a treatment condition was compared with the baseline). (D) Overexpression of cypin or cypinΔPDZ decreases the concentration of CNQX needed to affect overall activity in neuronal networks. (E) Organized activity as measured by bursting causes all networks to be sensitive to CNQX. Control networks treated with 5 μM CNQX are the only networks that do not experience a significant change compared with baseline. (F) The number of active electrodes was consistent across all conditions (*p < 0.05 and **p < 0.001 as determined by repeated-measures ANOVA followed by Tukey’s multiple comparisons test; *n* = 2 MEAs for each concentration for all conditions).

We found that networks overexpressing GFP (control networks) do not exhibit significant changes in the rate of spiking—a measure of overall activity—compared with baseline (no CNQX treatment) regardless of the concentration of CNQX treatment (representative raster plots shown in Figure 6A–6C; data quantified in Figure 6D). There is only a significant difference in spike rate between the 1 μM and 5 μM CNQX treatments for control networks. This lack of dose-dependent response might be due to compensatory mechanisms within the network and the effect of NMDAR-mediated synaptic transmission. However, in networks overexpressing cypin, all CNQX concentrations tested significantly reduced the overall spike rate. Interestingly, this effect is not dependent on cypin binding to PSD-95 or to its family members because networks overexpressing cypinΔPDZ also show similar decreases in spike rate at all concentrations of CNQX tested (Figure 6D). Similarly, all CNQX concentrations significantly reduced the burstlet rate—a measure of organized activity—for networks overexpressing cypin and for networks overexpressing cypinΔPDZ (Figure 6E). The bursting activity of control networks is also more sensitive to CNQX treatment than is spiking activity since control networks showed significantly decreased burstlet rates at most concentrations tested (Figure 6E). Moreover, while there were no significant differences observed between network types (i.e., control networks versus networks overexpressing cypin at a specific CNQX concentration), the average magnitude of burstlet rate decrease was greater for networks overexpressing cypin or networks overexpressing cypinΔPDZ than it was for control networks. This indicates that while the bursting activity of control networks is more sensitive to CNQX treatment than is spiking activity, the bursting activities of networks overexpressing cypin or networks overexpressing cypinΔPDZ are even more sensitive. Taken together, these results suggest that there is increased synaptic AMPAR expression and function in neuronal networks when cypin or cypinΔPDZ is overexpressed. Moreover, we found that the number of active electrodes was unchanged across conditions and treatments (Figure 6F), suggesting that these observed effects are due to changes in synaptic transmission and not due to a change in the number of neurons firing.

**Figure 7.**
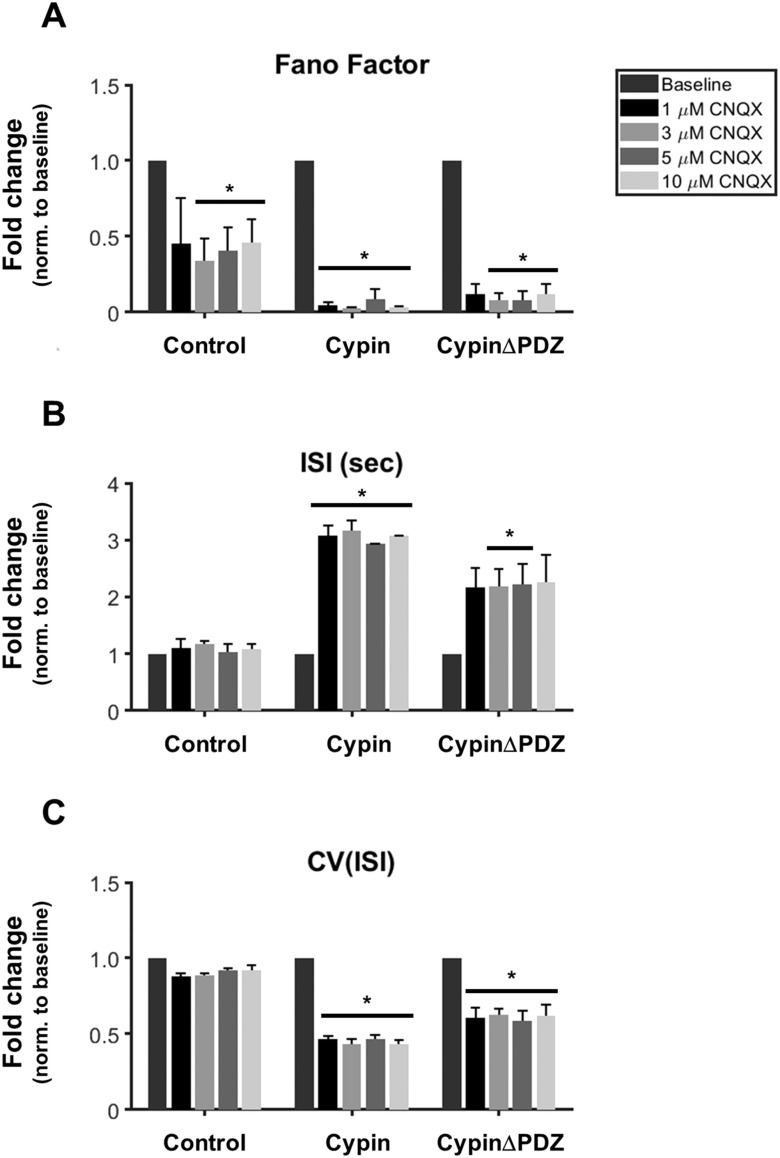
Spike variability is affected by CNQX treatment. (A) Spike count variability decreases after CNQX treatment in control networks, in networks overexpressing cypin, and in networks overexpressing cypinΔPDZ after treatment with 3, 5, or 10 μM CNQX compared with baseline. Fano factor is also significantly decreased in networks overexpressing cypin at 1 μM CNQX compared with baseline. (B) Cypin overexpression results in a significant increase in ISI regardless of CNQX concentration when compared with baseline, whereas cypinΔPDZ overexpression results in a significant increase in ISI only for the 3 and 5 μM CNQX treatment conditions when compared with baseline. For networks overexpressing cypinΔPDZ, the 1 and 10 μM CNQX treatments have *p* values of *p* = 0.063 and *p* = 0.074 when compared with baseline. (C) Cypin or cypinΔPDZ overexpression results in a significant increase in CV when compared with baseline, regardless of CNQX concentration (*p < 0.05 as determined by repeated-measures ANOVA followed by Tukey’s multiple comparisons test; *n* = 2 MEAs for each concentration for all conditions).

We also measured the spiking variability in these networks after CNQX treatment. We used the Fano factor (FF) as a measure of the variability of the spike count distribution as we have reported for similar networks (Rodriguez et al., 2018). Control networks exhibit a decrease in spike count variability after treatment with 3, 5, and 10 μM CNQX (Figure 7A), suggesting that the Fano factor decreases although spike rate remains unchanged (Figure 6D). FF significantly decreases when cypin or cypinΔPDZ is overexpressed at all concentrations of CNQX tested (Figure 7A), suggesting that AMPAR function influences spike variability. Furthermore, this analysis stresses the importance of studying spike variability in network responses as well as overall network activity to uncover differences that might otherwise go unnoticed.

In addition, networks overexpressing cypin or cypinΔPDZ show significant increases in the average interspike interval (ISI; Figure 7B) that are accompanied by significant decreases in the coefficient of variation (CV) of the ISI (Figure 7C) when compared with their respective baseline parameters. While networks overexpressing cypin demonstrate increased ISI compared with baseline regardless of CNQX concentration, networks overexpressing cypinΔPDZ only show significant increases in ISI compared with baseline for CNQX treatments of 3 and 5 μM (*p* = 0.063 and *p* = 0.074 for 1 and 10 μM, respectively). The changes to CV(ISI) mirror those seen in spike rate in Figure 6D: networks overexpressing cypin and networks overexpressing cypinΔPDZ both show significant increases in CV(ISI) regardless of CNQX concentration, whereas control networks are unaffected. Decreased spike rates after CNQX treatment in networks overexpressing cypin or cypinΔPDZ represent spikes that are temporally further apart; however, our data suggest that the variability in timing decreases and becomes more regular. In fact, the raw values of CV(ISI) demonstrate a baseline between 2.2–3.3, which decreases to near Poisson values (∼1) after CNQX exposure.

### Networks Overexpressing Cypin Delta PDZ Are More Resilient to Changes in Local Efficiency Induced by CNQX Treatment

The precise temporal resolution of MEA data allows the timing of spikes to be leveraged for functional connectivity analysis. We proceeded as follows: (1) we constructed matrices of pairwise cross-correlation between spike trains, which reflected neuron-to-neuron functional coupling; and (2) using the Brain Connectivity Toolbox (Rubinov & Sporns, 2010), we analyzed several network measures, including global efficiency, local efficiency, modularity (via community statistic Q), and number of communities for control networks, for networks overexpressing cypin, and for networks overexpressing cypinΔPDZ.

**Figure 8.**
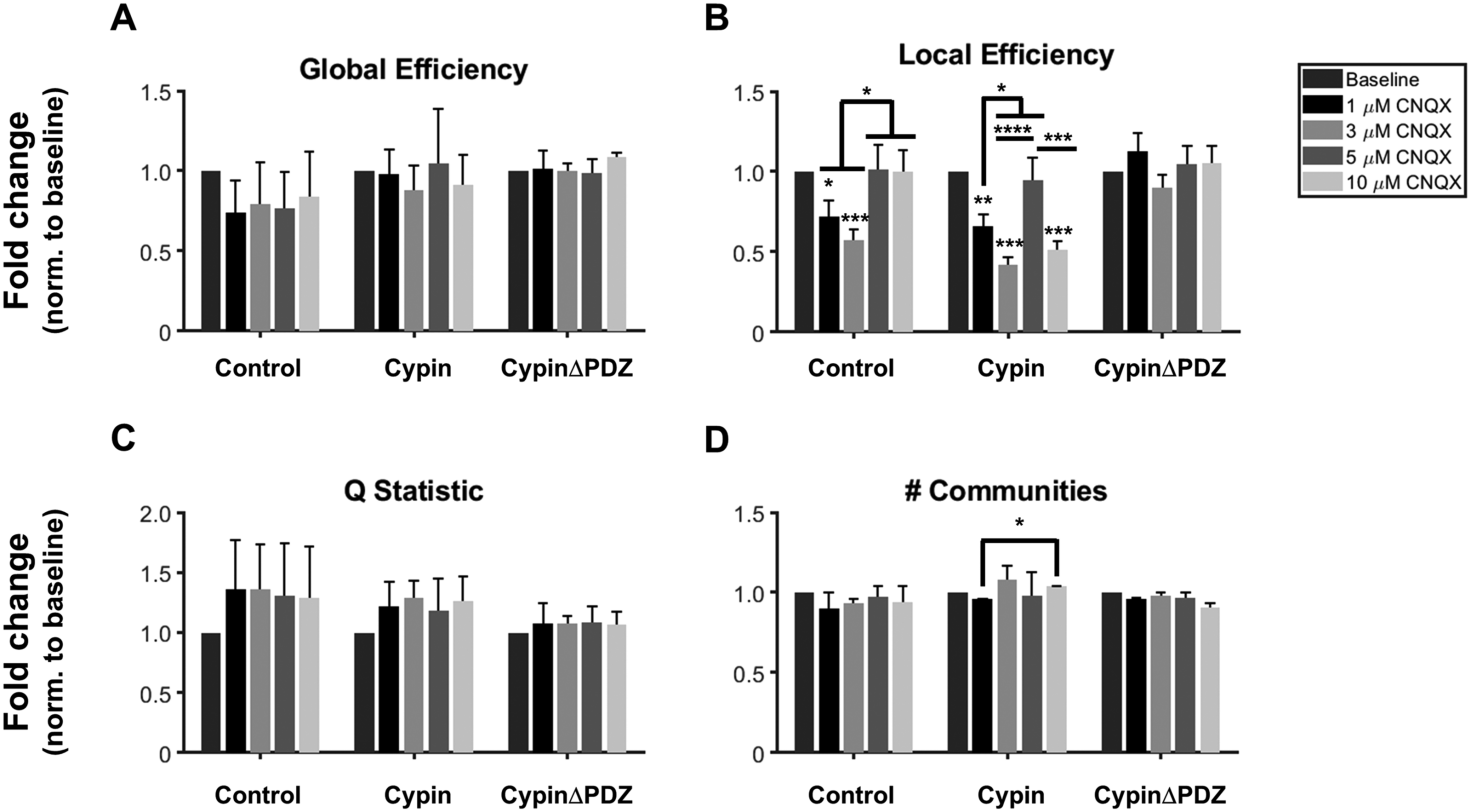
Networks of neurons overexpressing cypinΔPDZ show increased resilience to changes in local efficiency caused by CNQX. (A) Global efficiency of networks is not changed by cypin or cypinΔPDZ overexpression or by CNQX treatment. (B) Control networks and networks overexpressing cypin are similarly sensitive to CNQX-induced changes in local efficiency. On the contrary, networks overexpressing cypinΔPDZ do not demonstrate any significant changes in local efficiency. (C) Modularity of networks as measured by community statistic *Q* is not changed by cypin or cypinΔPDZ overexpression or by CNQX treatment. (D) The number of communities within networks does not change when compared with baseline for control networks, for networks overexpressing cypin, or for networks overexpressing cypinΔPDZ. The only significant change is observed between networks overexpressing cypin treated with 1 μM CNQX compared with those treated with 10 μM CNQX (*p < 0.05, **p < 0.01, ***p < 0.001, and ****p < 0.0001 as determined by repeated-measures ANOVA followed by Tukey’s multiple comparisons test; *n* = 2 MEAs for each concentration for all conditions).

While we observed no significant changes in global efficiency as a result of cypin overexpression or after CNQX treatment (Figure 8A), we observed a number of changes in efficiency at the local level (Figure 8B). Control networks demonstrated a significant decrease in local efficiency when treated with 1 and 3 μM CNQX when compared with baseline (no CNQX treatment). Interestingly, these changes disappeared at higher levels of CNQX (5 and 10 μM) when compared with baseline (Figure 8B). The changes to local efficiency for networks overexpressing cypin are similar: there are significant decreases in local efficiency when networks are treated with 1 and 3 μM CNQX and, additionally, when they are treated with 10 μM CNQX compared with baseline. The 5 μM CNQX treatment condition is not significantly different than the baseline. Even more surprisingly, networks overexpressing cypinΔPDZ were resilient to all changes in local efficiency caused by CNQX treatment.

Given that the local efficiency of in vitro networks seemed prone to changes from CNQX treatment, we expected to find corresponding changes in modularity or community structure. However, no significant changes to modularity were observed, likely due to high variability in control networks for all CNQX treatments (Figure 8C). There were also no significant differences in number of communities after CNQX treatment when compared with baseline for any of the conditions (Figure 8D). Only networks overexpressing cypin and treated with 1 μM CNQX were significantly different than those treated with 5 μM CNQX (with no difference between 1 or 5 μM CNQX compared with baseline).

### Average Spike Waveforms Are Altered by CNQX Treatment

**Figure 9.**
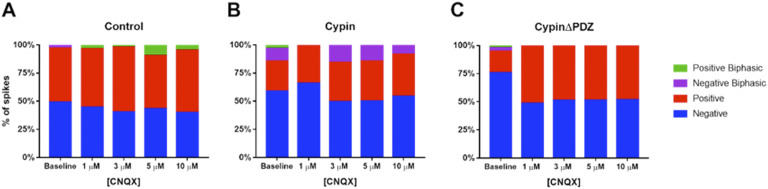
Spike waveforms after CNQX exposure. Distributions of spike shapes at baseline and after various CNQX treatments for (A) control networks, (B) networks overexpressing cypin, and (C) networks overexpressing cypinΔPDZ.

We previously performed spike sorting based on spike waveforms and reported that networks overexpressing cypin display certain spike shapes, specifically a larger proportion of nonnegative peaks that are not found in control networks or in networks overexpressing cypinΔPDZ (Rodriguez et al., 2018). To determine whether blocking AMPAR function with CNQX affects the shapes of detected spikes, we analyzed spike shapes at DIV14 before CNQX treatment and after treatment. Control networks exhibit subtle changes in the distribution of spike shapes after CNQX treatment, with greater positive biphasic waveforms at higher concentrations of CNQX (Figure 9A). Networks overexpressing cypin maintain the subset of negative biphasic spikes during the course of CNQX treatment, with the exception of networks treated with 1 μM CNQX (Figure 9B). Interestingly, networks overexpressing cypinΔPDZ display a complete loss of the subset of biphasic spikes observed before CNQX exposure and only exhibit a combination of negative and positive spikes that remains unchanged with CNQX treatment (Figure 9C). These data suggest that although CNQX treatment results in dramatic spike rate reduction and variability after cypin overexpression, the types of spikes that are produced and detected are largely unaffected by CNQX treatment.

### Simulated Cypin Overexpression Replicates In Vitro Findings

To get a clearer picture of how the neuronal network changes with cypin overexpression, we developed a computational model for connected neurons based on Masquelier and Deco (2013). Using the experimental observations following cypin manipulation, we developed the model to independently modify the strength of AMPAR conductance among neurons in the network as well as the relative density of connections among neurons. In addition, we explicitly modeled the enhanced presynaptic release that could occur from cypin overexpression. Our control network parameters were based on estimates from past models designed to match in vitro activity patterns observed in neuronal cultures (Masquelier & Deco, 2013).

**Figure 10.**
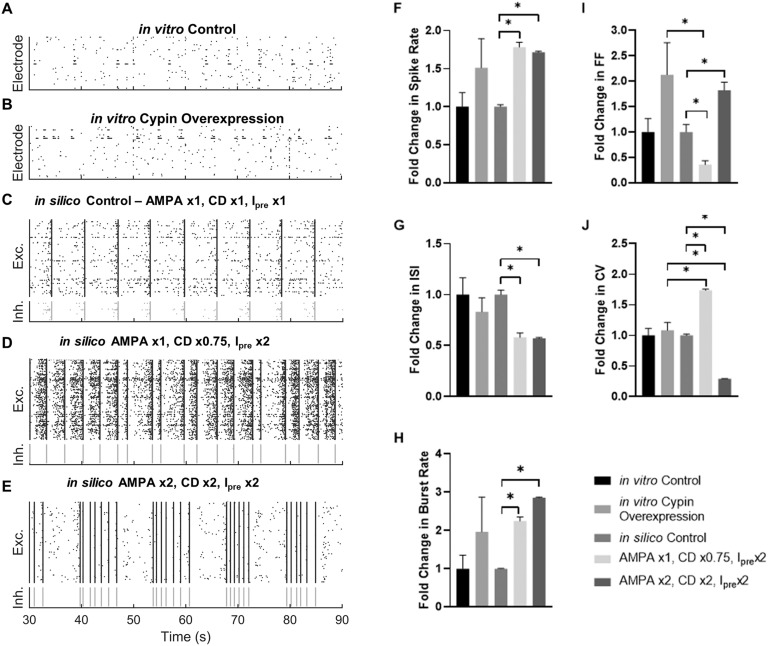
Modifying AMPA receptor conductance, connection density, and presynaptic current in silico recapitulates effects of in vitro cypin overexpression. Raster plots showing representative 60 sec of (A) in vitro control MEA recording; (B) in vitro cypin overexpression MEA recording; (C) control simulation where AMPAR conductance, connection density, and input noise are unmodified from Masquelier and Deco (2013); (D) simulation for cypin overexpression condition 1: AMPAR conductance is unmodified, connection density is multiplied by 0.75, and presynaptic current is doubled; and (E) simulation for cypin overexpression condition 2: AMPAR conductance is doubled, connection density is doubled, and presynaptic current is doubled. (F) Spike rate for in vitro and simulated cypin overexpression. Neither simulated cypin overexpression condition is significantly different from in vitro cypin overexpression. (G) Interspike interval (ISI) for in vitro and simulated cypin overexpression. Neither simulated cypin overexpression condition is significantly different from in vitro cypin overexpression. (H) Burst rate for in vitro and simulated cypin overexpression. Neither simulated cypin overexpression condition is significantly different from in vitro cypin overexpression. (I) Fano factor (FF) for in vitro and simulated cypin overexpression. Only simulated cypin overexpression condition 2 is not significantly different from in vitro cypin overexpression. (J) Coefficient of variation for in vitro and simulated cypin overexpression. Both simulated cypin overexpression conditions are significantly different from in vitro cypin overexpression. All values in the bar plots are normalized to their respective control; *p < 0.05 as determined by Welch’s correction for unequal variance (for comparisons of simulated cypin overexpression to in vitro cypin overexpression) and otherwise by uncorrected two-sample *t* test.

In general, cypin overexpression changes synaptic-scale phenomena and morphological features of the neural circuit. Some of these changes may offset each other—for example, cypin overexpression increases dendritic arborization (Akum et al., 2004; Rodriguez et al., 2018) but decreases spine density (Patel et al., 2018; Rodriguez et al., 2018)—leading to an uncertain net effect on circuit connectivity density. Likewise, new synaptic connections may not have the same synaptic strength as existing connections, leading to an uncertain effect on synaptic conductance. However, some observations point to a consistent change in modeling the effects, such as the increased frequency of mEPSCs. Therefore, we considered a broad range of parametric conditions to identify the most likely modeling conditions that matched experimental measurements of activity following cypin overexpression. Two conditions best matched our measured changes in vitro: (1) AMPAR conductance unchanged (AMPAx1), with a slight reduction in net connection density (CD × 0.75) and a twofold increase in presynaptic activity (I_pre_ × 2) (Figure 10D), and (2) a twofold increase in AMPAR conductance (AMPA × 2) and net connection density (CD × 2) with a commensurate increase in presynaptic activity (I_pre_ × 2) (Figure 10E). Relative to the control case (Figure 10C), both of these simulated cypin overexpression networks showed increased spike rate (*p* < 0.0001 for both, two-sample *t* test; Figure 10F) much like that observed with in vitro cypin overexpression (Figure 10B) relative to the in vitro control case (*p* > 0.5 for both simulation conditions relative to in vitro cypin overexpression, two-sample *t* test with Welch’s correction for unequal variance; Figure 10A; summarized in Table 1). There was a commensurate decrease in ISI (*p* < 0.01 for both relative to control, two-sample *t* test; Figure 10G) for both simulated cypin overexpression networks, as observed for in vitro cypin overexpression (*p* > 0.09 for both relative to in vitro cypin overexpression, two-sample *t* test with Welch’s correction). Likewise, there was a corresponding increase in burst frequency that occurred for both simulations of cypin overexpression relative to control networks (*p* < 0.001 for both, two-sample *t* test; Figure 10H) that paralleled what we found in in vitro cypin overexpression networks (*p* > 0.3, two-sample *t* test with Welch’s correction; Figure 10H). However, for the first set of in silico cypin overexpression conditions (AMPA ×1, CD × 0.75, I_pre_ × 2), the FF significantly decreased relative to the control condition (*p* = 0.005, two-sample *t* test; Figure 10I), while for the second set of in silico conditions (AMPA × 2, CD × 2, I_pre_ × 2), the FF significantly increased (*p* = 0.005, two-sample *t* test; Figure 10I) and more closely matched that of the in vitro cypin overexpression case (*p* = 0.02 for in silico cypin overexpression condition 1 vs. *p* = 0.65 for in silico cypin overexpression condition 2, two-sample *t* test with Welch’s correction; Figure 10I). For the CV of the ISI, the first set of in silico cypin overexpression conditions were significantly higher than those for the in silico control network (*p* < 0.0001; two-sample *t* test; Figure 10J) and significantly lower for the second set of in silico cypin overexpression conditions (*p* < 0.0001; two-sample *t* test; Figure 10J) and neither simulated condition matched those for the in vitro cypin overexpression findings (*p* < 0.001 for both, two-sample *t* test with Welch’s correction; Figure 10J).

**Table 1.** Simulated in silico cypin overexpression reproduces changes found in vitro.

Condition	Spike rate	Interspike interval	Burst rate	Fano factor	Coefficient of variation	Global efficiency	Number of communities	Community statistic *Q*
In vitro cypin overexpression	1.5 ± 0.4	0.8 ± 0.1	2.0 ± 0.9	2.1 ± 0.6	1.1 ± 0.1	1.0 ± 0.07	1.1 ± 0.07	0.9 ± 0.05
In silico cypin overexpression 1: AMPA × 1, CD × 0.75, I_*pre*_ × 2	1.8 ± 0.04	0.6 ± 0.04	2.2 ± 0.1	0.4 ± 0.09	1.7 ± 0.02	0.8 ± 0.002	2.3 ± 0.1	0.8 ± 0.01
In silico cypin overexpression 2: AMPA × 2, CD × 2, I_*pre*_ × 2	1.7 ± 0.01	0.6 ± 0.01	2.9 ± 0.02	1.8 ± 0.2	0.3 ± 0.004	1.3 ± 0.007	0.9 ± 0.1	1.1 ± 0.03

*Note*. Reported values are normalized to the control case with endogenous cypin expression, AMPA receptor conductance, connection density, and presynaptic current. Data are reported as mean ± *SEM*.

**Figure 11.**
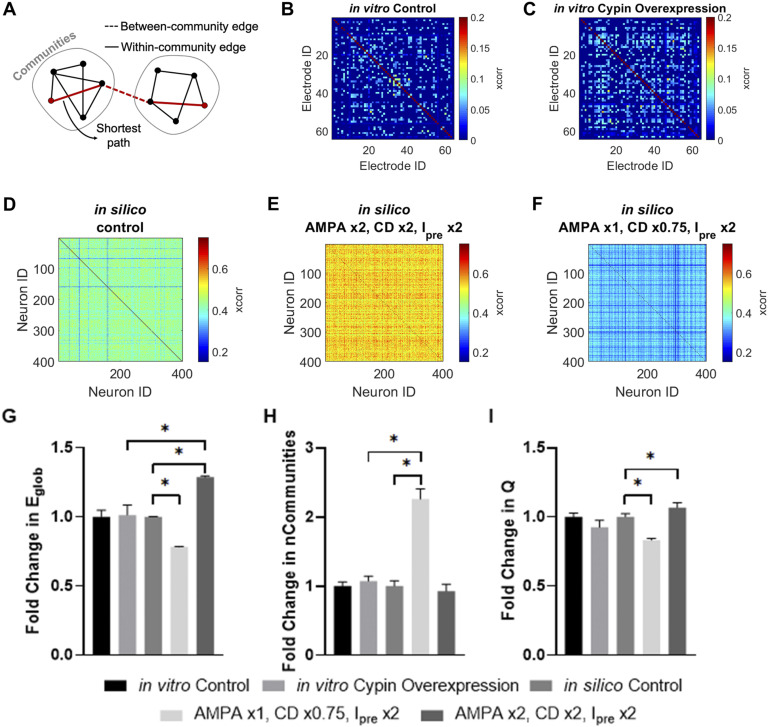
Cypin overexpression maintains network functional organization. (A) Overview of network measures. Global efficiency is computed based on the average inverse shortest path length in the network. Communities are identified by maximizing the within-community connectivity while minimizing between-community connectivity. Community statistic *Q* reflects how tightly knit the communities are, with a higher *Q* reflecting denser within-community connectivity relative to between-community connectivity. (B–F) Representative functional connectivity matrix for (B) in vitro control; (C) in vitro cypin overexpression; (D) in silico control; (E) in silico cypin overexpression condition 1: AMPAR conductance is unmodified, connection density is multiplied by 0.75, and presynaptic current is doubled; and (F) in silico cypin overexpression condition 2: AMPAR conductance is doubled, connection density is doubled, and presynaptic current is doubled. (G) Fold change in global efficiency (E_glob_) shows no change between in vitro cypin overexpression and its control that is reproduced by in silico cypin overexpression condition 1 but not condition 2. (H) Fold change in number of communities shows no change for in vitro cypin overexpression relative to its control, which is reproduced by cypin overexpression condition 2, but not condition 1. (I) Fold change in community statistic *Q* shows no difference between in vitro cypin overexpression relative to its control, or compared with the fold change in in silico cypin overexpression condition 1 or 2. All values in the bar plots are normalized to their respective control; *p < 0.05 as determined by two-way ANOVA with Tukey’s post hoc test.

To further explore which simulated cypin overexpression condition best matched in vitro cypin overexpression, we examined the network properties of the simulated networks (Figure 11A). We first constructed functional connectivity matrices for the in vitro control (Figure 11B), in vitro cypin overexpression (Figure 11C), in silico control (Figure 11D), in silico cypin overexpression condition 1 (Figure 11E), and in silico cypin overexpression condition 2 (Figure 11F) based on the pairwise cross-correlation between spike trains in each culture or simulation. Using those functional connectivity matrices, we sought to investigate communication and community structure in the network. As a result, we computed the global efficiency (Figure 11G) to measure ease of information transfer in the network, number of communities (Figure 11H) to measure possible functional states the network could inhabit, and community statistic *Q* (Figure 11I) to measure how tightly bound the community states were (network analysis summarized in Table 1). We found that there were no observed changes in network structure for the in vitro networks (Figure 11G–11I). Relative to the simulations, in vitro cypin overexpression was indistinguishable from in silico cypin overexpression condition 1, while in silico cypin overexpression condition 2 showed an increase in global efficiency (Figure 11G). Although there was a significant increase in number of communities for in silico cypin overexpression condition 1 that did not match in vitro cypin overexpression, in silico cypin overexpression condition 2 did not demonstrate a change in number of communities, thus matching the in vitro case (Figure 11H). For changes in the community statistic *Q*, neither in silico cypin overexpression condition significantly differed from the in vitro networks overexpressing cypin (Figure 11I).

Together, these in silico cypin overexpression conditions, evaluated by both spike train analysis and network analysis, represent reasonable approximations of our in vitro observations, and we pursued both in parallel to evaluate how cypin overexpression might affect signal transmission in a neural network.

### Simulated Cypin Overexpression Decreases Signal Fidelity

**Figure 12.**
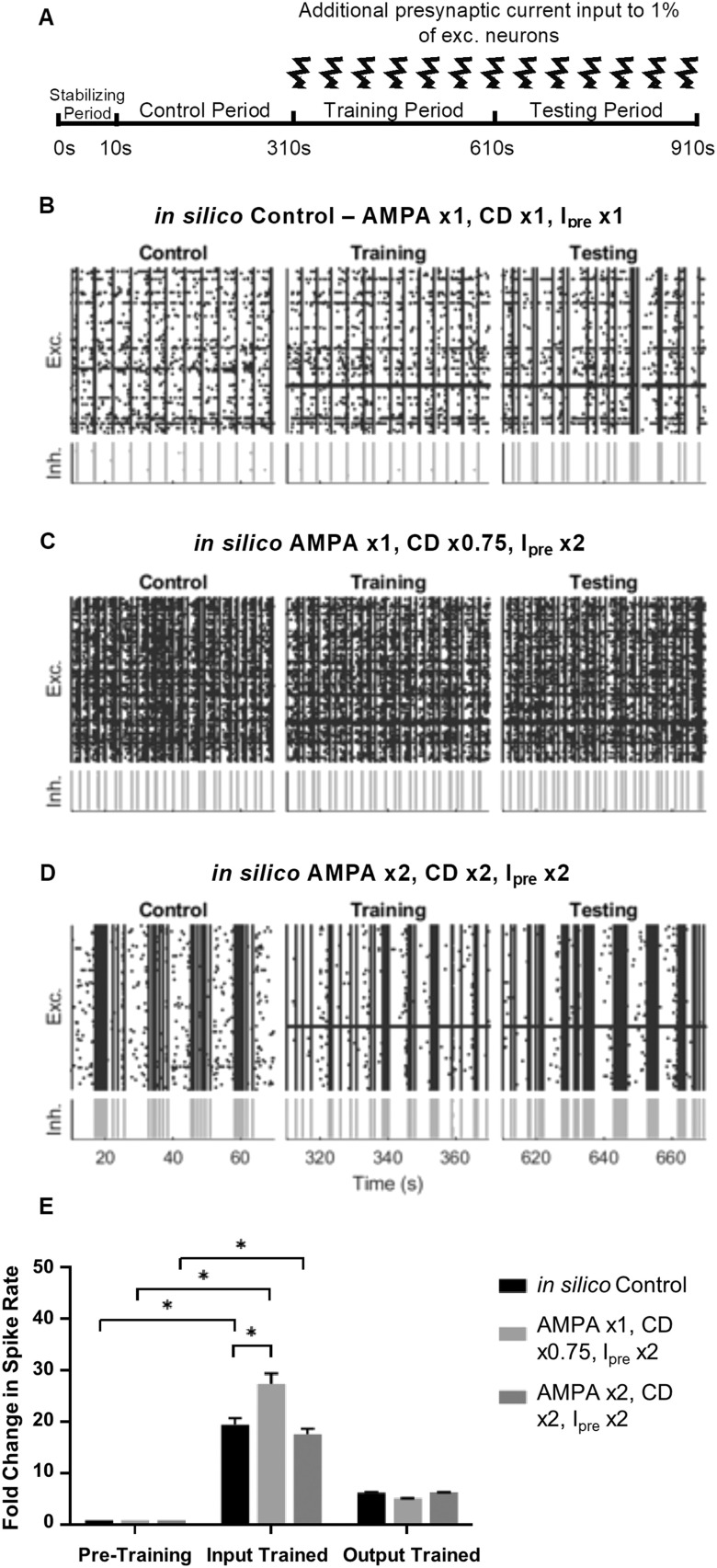
Signal fidelity decreases for in silico cypin overexpression condition 1 but does not change for in silico cypin overexpression condition 2. (A) Simulation design for investigating conditioned response to stimulus. Simulations begin with a 10-sec stabilization period, 300 sec of control period, 300 sec of training wherein the external input current is doubled for 1% of excitatory input neurons and output neurons are conditioned, then 300 sec of testing where the response of the output neurons to the conditioned stimulus is evaluated. (B) Raster plot for the in silico control condition where AMPA receptor conductance, connection density, and input noise are unmodified, but 1% of excitatory neurons receive double presynaptic input current from 310 sec onward. (C) Raster plot for conditioned response to external stimulus for in silico cypin overexpression condition 1 where AMPA receptor conductance is unmodified, connection density is multiplied by 0.75, and I_pre_ is doubled. (D) Raster plot for conditioned response to external stimulus for in silico cypin overexpression condition 2 where AMPA receptor conductance is doubled, connection density is doubled, and I_pre_ is doubled. (E) Fold change in spike rate for in silico control case and cypin overexpression conditions 1 and 2 for input and output neurons after training. All conditions show a significant change in spike rate following presynaptic current input (*p* < 0.0001 for all conditions, two-sample *t* test). The fold change in the spike rate for input-trained neurons is significantly different for in silico cypin overexpression condition 1 compared with in silico control (*p* < 0.0001, two-sample *t* test), but not for in silico cypin overexpression condition 2. There was no significant difference in the fold change in spike rate for the output neurons across conditions. For the control case, the signal fidelity between output and input neuron spiking is 0.33 ± 0.002. For in silico cypin overexpression condition 1, the signal fidelity is 0.19 ± 0.001, which is significantly different from the in silico control case (*p* < 0.01, two-sample *t* test). For in silico cypin overexpression condition 2, however, the signal fidelity is 0.36 ± 0.003 and not significantly different from in silico control (*p* = 0.29, two-sample *t* test); *p < 0.05.

To test how cypin overexpression affects the fidelity of signal transmission in the simulated network, we simultaneously doubled presynaptic current input for 1% of the excitatory neurons (termed input neurons) and, after a predefined training period, recorded how the firing rate changed in the rest of the simulated network (termed output neurons) in the testing period (Figure 12A). In the in silico control condition (Figure 12B), injecting this current into a subset of neurons led to a significant increase in the firing rate of target (input) neurons (relative fold increase of 19.5 ± 3.0 with stimulation; *p* < 0.0001; Figure 12E) compared with the pretraining level. This injection of external current also led to an increase in the firing rate of the remaining excitatory neurons in the network compared with pretraining levels (relative fold increase of 6.3 ± 0.3; *p* < 0.001; Figure 12E), although this firing rate was less than the increase observed in the input neurons. As one measure of signal transmission in the simulated network, we found the ratio of the firing rate between the output and input neurons, termed signal fidelity, changed significantly after training (1.02 ± 0.01, before training, vs. 0.33 ± 0.002, after training; *p* < 0.0001, two-sample *t* test), demonstrating the ability of the circuit to modify its output in response to a specific input.

The two most likely modeling scenarios (Figure 12C and 12D) that best mimic the experimental physiological changes in cypin overexpression show differential effects of signal transmission in the simulated circuit. If we consider a model that matches firing rate and ISI only (Figure 12C), we find that the network shows a significant increase in firing rate of the input neurons after training (relative fold increase of 27.5 ± 4.6, *p* < 0.0001; Figure 12E) and in output neurons after training (relative fold increase of 5.3 ± 0.06, *p* < 0.0001; Figure 12E). The signal fidelity was significantly different in this network compared with the control network (SF = 0.19 ± 0.001, *p* < 0.001, two-sample *t* test; data not shown) due to a significant increase in spike rate in the input neuron population (*p* < 0.0001, two-sample *t* test). In contrast, in a model that matches the firing rate, ISI, and FF after cypin overexpression (Figure 12D), we observe that the network shows no change in signal fidelity relative to control (SF = 0.36 ± 0.003, *p* = 0.29, two-sample *t* test; data not shown). The key difference between these two simulations of cypin overexpression in relation to spiking activity is the difference in FF; other characteristics of the two circuits were not different from each other.

**Figure 13.**
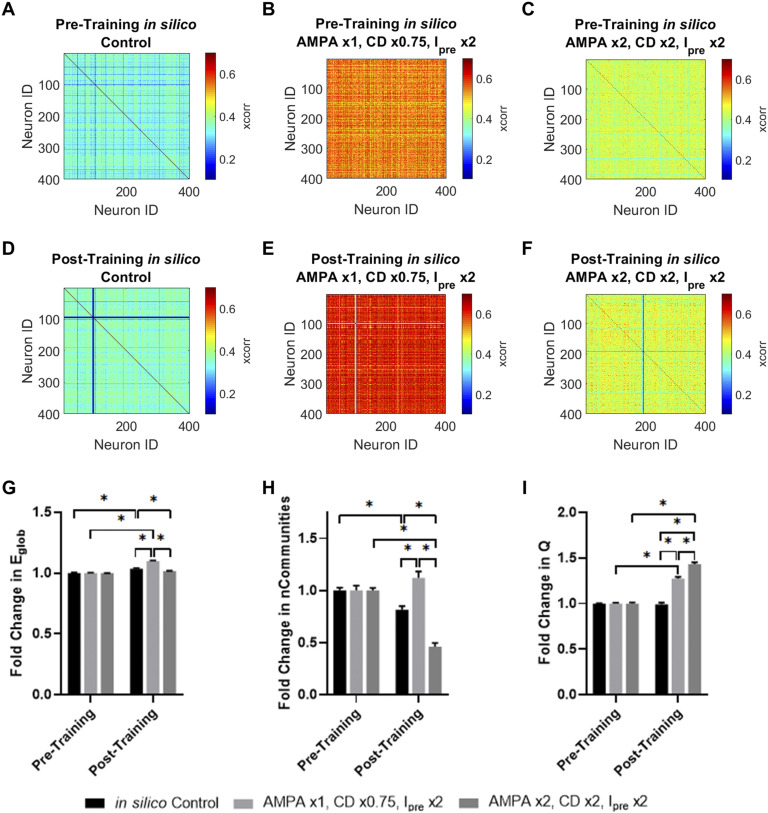
In silico cypin overexpression networks reorganize following signal entrainment. (A–C) Pretraining functional connectivity matrices for (A) in silico control; (B) in silico cypin overexpression condition 1: AMPAR conductance is unmodified, connection density is multiplied by 0.75, and presynaptic current is doubled; and (C) in silico cypin overexpression condition 2: AMPAR conductance is doubled, connection density is doubled, and presynaptic current is doubled. (D–F) Posttraining functional connectivity matrices for (D) in silico control, (E) in silico cypin overexpression condition 1, and (F) in silico cypin overexpression condition 2. (G) Fold change in global efficiency (E_glob_) normalized to pretraining period. (H) Fold change in number of communities normalized to pretraining period. (I) Fold change in community statistic *Q* normalized to pretraining period (*p < 0.05 as determined by two-way ANOVA with Tukey’s post hoc test).

We wanted to further explore how the simulated circuit itself remodeled in response to signal input. To that end, we created functional connectivity networks for the pretraining and posttraining in silico networks (Figure 13A–13F) and calculated the fold change in global efficiency (Figure 13G), number of communities (Figure 13H), and community statistic *Q* (Figure 13I). We found that all three in silico networks were distinct from each other after training for all three network measures (Figure 13G–13I), suggesting that each network remodels in a way entirely distinct from the others. The in silico cypin overexpression condition 1 demonstrated enhanced global efficiency, an increase in number of communities, and an increase in the community statistic (Figure 13G–13I), suggesting a network in which there is increased overall connectivity with creation of new, more tightly knit communities. In contrast, in silico cypin overexpression condition 2 exhibits no change in global efficiency, a marked decrease in number of communities, and a large increase in the community statistic (Figure 13G–13I), suggesting that the network consolidates into fewer, very tightly knit communities after stimulation. From a networks perspective, neither simulation of cypin overexpression matches the in vitro cypin overexpression more faithfully, but both provide insight into how networks respond to changes in network-wide activity.

## DISCUSSION

In this work, we report that cypin regulates PSD-95 localization and affects synaptic transmission at the single cell and network levels. Overexpression of cypin decreases PSD-95 content at the synapse, and this decrease is dependent on cypin binding to PSD-95. Cypin overexpression also increases synaptic transmission, supporting a role for increased synaptic AMPA receptors in cypin action. Specifically, in vitro networks that overexpress cypin show increased sensitivity to the AMPA receptor antagonist CNQX. Surprisingly, overexpression of a mutant cypin lacking the PDZ-binding motif (cypinΔPDZ), and hence lacking PSD-95 binding, results in similar changes to network activity and spike variability as does wild-type cypin. Taken together, our data suggest a role for cypin in AMPA receptor function that is independent of PSD-95. When we incorporated these findings into a computational model of a neuronal network, mimicking these changes following cypin manipulation suggests that cypin may significantly influence information content and the fidelity of signal transmission in a neuronal circuit.

These results are surprising given our findings regarding the observed PDZ-binding-dependent decrease in synaptic PSD-95 protein levels with cypin overexpression. However, they are consistent with our previous results that overexpression of cypin alters neuronal morphology and function via distinct mechanisms, whereby cypin binding to PSD-95 is dispensable for regulating dendritic morphology but is important for shaping network variability (Rodriguez et al., 2018). Importantly, when we performed network-level analysis, we found that the cypinΔPDZ mutant significantly influences the information content of the circuit, but these changes are not observed under baseline conditions. Rather, they become evident when the network is challenged with increasing concentrations of the competitive AMPA receptor antagonist CNQX. Functional defects that result from PSD-95 deletion in mice are restricted to a subset of synapses (Beique et al., 2006), and thus, it is possible that the synaptic defects we observed with cypin overexpression, which decreases synaptic PSD-95 localization, may affect a small population of synapses. Moreover, it is possible that overexpression of cypinΔPDZ also causes changes to a small population of synapses that are not observable under baseline conditions and instead become evident when the network is presented with a challenge, that is, CNQX.

We observed that the overall spike rate of networks overexpressing cypin, but not control networks, is dramatically decreased after treatment with the AMPAR antagonist CNQX at all concentrations tested, suggesting that cypin overexpression causes an increase in functional AMPARs in these networks. Our data thus suggest a mechanism distinct from cypin-mediated decreases in synaptic targeting of PSD-95. Previous findings (Yudowski et al., 2013) combined with the fact that spike rates of control networks are resistant to CNQX suggest that AMPAergic synaptic upscaling may be triggered in the control networks in response to this treatment as a compensatory mechanism (Fong et al., 2015). In cypin and cypinΔPDZ networks, the increase in functional AMPA receptors may prevent homeostatic upscaling. It should be noted that we did not analyze global bursting (network-wide bursts) or synchronization behavior in these networks because suppression of activity resulted in extremely low rates of global bursting events, and many times global bursting was not present (see raster plots in Figure 6A–6C).

It is possible that the observed changes to neuronal physiology, particularly mEPSCs and local efficiency, are due to changes to presynaptic vesicle release. We previously reported that snapin binds to cypin, resulting in decreased dendritic branching (Chen et al., 2005). Snapin is a SNAP-25/23 interacting protein that, at presynaptic sites, binds the SNARE complex through SNAP-25 (Ilardi et al., 1999). Studies investigating the effects of snapin manipulation on neuronal electrophysiology and vesicle release dynamics show that snapin deficiency induces a decrease in mEPSC frequency and kinetics in vivo (Pan et al., 2009). This deficiency also causes a widespread desynchronization of presynaptic vesicle exocytosis and subsequent desynchronization of EPSCs (Pan et al., 2009). It is possible then that cypin binding to snapin increases its trafficking to presynaptic sites, or modulates snapin activity, leading to increased vesicle priming and release, effectively increasing mEPSC frequency.

Our computational model is closely aligned with our embryonic rat hippocampal culture model and has key features of synaptic receptors found in vitro, the typical ratio of excitatory and inhibitory neurons for in vitro networks, and the appropriate parameters for baseline presynaptic release and postsynaptic activity (Capone et al., 2018; Fardet et al., 2018; Tyukin et al., 2019). However, our model does not explicitly weight synaptic inputs by where they are located on the dendritic arbor (Hausser, 2001). Future work that simulates both cypin’s effects on the dendritic arbor and on network activity would be interesting since experimental work demonstrated that excitatory synaptic input is greater at dendrite branch origins compared with branch ends (Katz et al., 2009). We are, nevertheless, encouraged by the ability of this simplified model to match many of the circuit-level parameters that change with cypin overexpression in vitro and attribute differences between in vitro and in silico data to the spatial and temporal filtering at each electrode.

One clear change in the circuit behavior was a change in the bursting behavior, with cypin overexpression increasing burst frequency in vitro. In its most extreme form, persistent bursting may be the basis for aberrant signaling, such as in epilepsy (reviewed in McCormick & Contreras, 2001). At nonpathological levels of bursting, coordinated activations of entire networks could serve to relay information from one brain area to another (as reviewed in Bressler & Kelso, 2001; Haber & Calzavara, 2009, and described by Hwang et al., 2017; Tyagi, 2015; Zingg et al., 2014, and others). In addition, these oscillations within one area may synchronize with other brain regions, leading to the transient activation of brain networks that might be critical for performing cognitive tasks (Harmony, 2013; Nyhus & Curran, 2010). Both simulation approaches modeled to match in vitro observations show an increase in bursting frequency, suggesting that cypin overexpression may facilitate this information relay process that is coordinated among brain regions.

Another circuit-level change induced by cypin is the change in information content. Of the many measures used to describe information in neural systems, firing rate is commonly associated with information in networks, whether at the single neuron or circuit levels (Azarfar et al., 2018; Gallistel, 2017; Ponulak & Kasinski, 2011; Rozeske & Herry, 2018). Our simulations and experimental observations suggest that increasing cypin levels will increase firing rates, thus increasing the information content. An increase in firing rate may also accompany a change in the number and diversity of cells that fire in sequence with each other, known as engrams (Mayford, 2014). In combination, these changes suggest that cypin overexpression can increase the number and complexity of information patterns within a circuit and can facilitate the transfer of this information across broader networks in the brain.

A final component to consider from our simulations is the effect of in silico cypin overexpression on signal entrainment in a network. In in silico control networks, stimulating an input layer of neurons caused significantly higher firing rates in the output layer neurons while only slightly changing network parameters (slightly raising global efficiency, decreasing number of communities, no effect on modularity). Therefore, our stimulation protocol allowed us to manipulate one aspect of information in the output layer (firing rate) without substantially affecting network structure. Intriguingly, both simulations of cypin overexpression led to marked changes in both information and structure. Both models of cypin overexpression strengthened connections within communities, implying that cypin overexpression would confer some level of resilience to the network (Sporns & Betzel, 2016). This prediction is supported by the in vitro data demonstrating that local efficiency of networks overexpressing cypinΔPDZ are more resilient to CNQX treatment than are control networks or networks overexpressing cypin.

Neither model substantially affected global efficiency relative to control networks, which may be advantageous because of the important role that global efficiency can play for relaying signals throughout the brain. The most striking difference between the two models of cypin overexpression is the number of communities created in the output layer neurons after entrainment. One model of cypin overexpression shows a significant decline in the number of communities after entrainment, presenting a network that is more reliable and potentially more resilient but that is less structurally flexible. The importance of community number is suggested in several studies of larger scale brain networks, where an increased number of communities is associated with positive affect (Betzel et al., 2017). In this framework, cypin overexpression may have important adverse consequences on the function of the network. In comparison, the alternative model of cypin overexpression shows a slight increase in the number of communities, again without adversely affecting global efficiency, and this more flexible structure may facilitate information transfer to other connected networks. Alternatively, these networks may achieve more complex network states, given the increase in the number of individual communities. In combination, these simulations raise the intriguing possibility that cypin may play a role in cognitive processes, whether at the local circuit level to remodel circuits after injury or at a more global level to coordinate the activation of different brain regions during cognitive tasks.

Overall, our data demonstrate the effect that cypin will have on both the extent and strength of connectivity within a neural circuit as well as the influence it will have on passing information through a circuit. The role of cypin across these different length scales indicates that it is a key component in remodeling and shaping circuit activity, with intriguing implications on how it can facilitate the information transfer process needed during cognition as well as the repair of the brain in acute and chronic neurodegenerative disorders. Rather than considering only the important molecular roles that cypin can play as a mediator of spine density and maturity, these data point to a much broader and influential role for cypin in microcircuits. In combination with the prominent role of cypin in purine metabolism, our work demonstrates that cypin could be a key therapeutic target for manipulating and reconstructing diseased circuits, rather than only focusing on its role in shaping local connectivity points among neurons.

## MATERIALS AND METHODS

### Antibodies and Reagents

Rabbit polyclonal antibody raised against cypin has been previously described (Firestein et al., 1999). Either mouse monoclonal PSD-95 antibody from Thermo Scientific (Waltham, MA) or rabbit anti-PSD-95 from Abcam (Cambridge, MA) was used for immunoblotting. Rabbit anti-synaptophysin was purchased from Zymed (San Francisco, CA). The 5′ end mutated U1 snRNA constructs have also previously been described (Akum et al., 2004). In particular, we used the U1-3′UTR snRNA and the rescue plasmid characterized in our previous work (Akum et al., 2004).

### Cell Culture for Immunocytochemistry and Biochemistry

Hippocampi were dissected from Sprague-Dawley rat embryos at 18 days of gestation as we described previously (O’Neill et al., 2018; O’Neill, Kwon, Donohue, & Firestein, 2017; Rodriguez et al., 2018; Swiatkowski et al., 2018). The hippocampi were mechanically dissociated, and cells were plated on 12-mm glass coverslips or in 6-well plates coated with 0.5 mg/ml poly-D-lysine (PDL; Sigma). Cultures were maintained in Neurobasal medium supplemented with B27 and GlutaMAX (all from ThermoFisher) at 37°C and 5% CO_2_. All animal experiments were approved by the Rutgers University Institutional Animal Care and Use Committee.

### Lentiviral Particle Production and Transduction

The lentiviral plasmids used were reported in Rodriguez et al. (2018) and include shRNAs against glutathione S-transferase as control (Rodriguez et al., 2018). Briefly, lentiviral particles were generated from HEK293TN cells (ATCC) transfected with one lentiviral plasmid, the packaging plasmid psPAX2, and the envelope plasmid pMD2.G (VSV-G). Viral particles were concentrated from the medium using PEG-it virus precipitation solution (System Biosciences). At the indicated day in vitro (DIV), hippocampal cultures were transduced with lentiviral supernatant, and half of the culture medium was changed 36 hours later and every two days after. Experiments were performed in sister cultures like those reported in Rodriguez et al. (2018) and Swiatkowski et al. (2018) where overexpression and knockdown were confirmed.

### Subcellular Fractionation

On DIV21, neurons from two wells of a 6-well plate for each condition were scrape-harvested into 150 μl of homogenization buffer (HB; 320 mM sucrose, 4 mM HEPES, 1 mM EGTA, 1 mM PMSF). Subcellular structures were fractionated as we previously described (Chen & Firestein, 2007; Chen et al., 2005; Patel et al., 2018). The cell membranes were disrupted by passing the cells through a 25½-gauge needle approximately 10 times. The homogenate was centrifuged at 1,000 × *g* for 10 minutes at 4°C. The supernatant (S1) was collected and centrifuged at 12,000 × *g* for 15 minutes at 4°C. The pellet (P2) was resuspended and washed in 100 μl of HB and centrifuged at 13,000 × *g* for 15 minutes at 4°C. The resulting pellet (P2′), representing a crude synaptosomal fraction, was lysed by osmotic shock and homogenized by pipetting up and down multiple times. The homogenate was spun at 33,000 × *g* for 20 minutes at 4°C to yield supernatant LS1 and pellet LP1 (heavy membranes). LS1 was spun at 251,000 × *g* for 2 hours at 4°C. The resulting supernatant (LS2) contained soluble proteins, while the pellet (LP2) contained synaptic vesicle proteins. Each fraction was stored at −20°C in protein loading buffer.

### Western Blot Analysis

On DIV21, cultured neurons were scraped and harvested into RIPA buffer (50 mM Tris-HCl pH 7.4, 150 mM NaCl, 1 mM EGTA, 1% NP-40, 0.25% sodium deoxycholate, 0.1% SDS, 1 mM PMSF) and lysed with a 25½-gauge needle. The lysates were spun for 15 minutes at 13,000 × *g* at 4°C, and the supernatant (which contains proteins) was collected. Proteins were resolved by 10% SDS-polyacrylamide gel electrophoresis, and proteins were transferred to polyvinylidene difluoride membrane (Millipore). The blot was probed with the indicated antibodies and scanned, and band intensities were quantified using ImageJ software (National Institutes of Health).

### Immunocytochemistry and Assessment of Synaptic Clustering

Neurons were transfected with the aforementioned constructs (GFP, cypin, and cypinΔPDZ) at DIV10 using Effectene (Qiagen, Hilden, Germany) and fixed with ice-cold methanol for 15 minutes on DIV12. Neurons were immunostained with the following primary antibodies: mouse anti-PSD95 (1:200), rat anti-GFP (1:1,000), and rabbit anti-synaptophysin (1:500).

To quantify PSD-95 clusters, images of neurons were captured on an Olympus Optical (Tokyo, Japan) IX50 microscope with ×60 oil objective using a constant gain and exposure time, filling the 12-bit dynamic range. Cluster outlines were calculated for fluorescent signals that were two standard deviations above unlocalized baseline using a macro written for ImagePro (Charych et al., 2006). Cluster area was measured for each outlined cluster. Cluster number was calculated by counting the average number of clusters per square micrometer of dendritic area.

### Electrophysiology

Whole-cell patch-clamp recordings were obtained from the soma of hippocampal neurons as described previously (Hernandez et al., 2016) on DIV21. The external solution contained the following (in mM): 140 NaCl, 5 KCl, 2 CaCl_2_, 2 MgCl_2_, 10 HEPES, and 10 glucose (pH 7.4 adjusted with NaOH; 290–310 mOsmol). Recording electrodes (3–5 MΩ) contained a K^+^-based internal solution composed of the following (in mM): 126 K-gluconate, 4 KCl, 10 HEPES, 4 ATP-Mg, 0.3 GTP-Na_2_, 10 phosphocreatine, and 10 QX-314 bromide (pH 7.2; 280–300 mOsmol). Action potentials were blocked with 1 μM tetrodotoxin (Tocris, R & D Systems) to record miniature excitatory postsynaptic currents (mEPSCs). The membrane potential was held at −70 mV throughout all experiments. Data were amplified and filtered at 2 kHz by a patch-clamp amplifier (Multiclamp 700B), digitalized (DIGIDATA 1440A), stored, and analyzed by pCLAMP (Molecular Devices). Data were discarded when the input resistance changed >20% during recording.

### Cell Culture on Microelectrode Arrays

Microelectrode arrays (MEAs; Multi Channel Systems, Germany) were coated with 0.5 mg/ml PDL (Sigma) for at least 1 hour at 37°C, washed three times with sterile water, and then coated with 10 μg/ml laminin (Sigma) for at least 30 minutes at 37°C. Cultures were established at a density of 1 ×10^6^ cells/MEA and maintained in NbActiv4 medium (BrainBits, LLC) at 37°C and 5% CO_2_. Half medium changes were performed every other day.

### CNQX Treatment

Increasing amounts of the competitive AMPAR antagonist CNQX, ranging between 1 and 10 μM and diluted in recording solution (Kutzing et al., 2011, 2012; Rodriguez et al., 2018), were added to the cultures, and activity was recorded for 5 minutes after equilibration was reached. After each recording, the cultures were washed twice with fresh culture medium. The networks recovered for at least 15 minutes between each CNQX treatment and recording.

### MEA Recordings

Recordings were performed as described in Rodriguez et al. (2018). Briefly, neurons were cultured on standard MEAs containing 59 recording and 1 internal reference planar electrodes, each with a 10 μm diameter and an interelectrode spacing of 200 μm (60MEA200/10iR-Ti-gr; Multi Channel Systems, Germany). Baseline recordings were performed on DIV10 followed by lentiviral transduction with GFP, cypin, or cypinΔPDZ constructs, and recordings were taken again on DIV14 with increasing concentrations of CNQX. Each MEA was covered with a semipermeable lid (ALA MEA-MEM; ALA Scientific) during handling and recordings to prevent contamination from airborne pathogens.

Spontaneous electrical signals were recorded for 5 minutes using the data acquisition commercial software MC_Rack (Multi Channel Systems) as we have previously described (Kutzing et al., 2011, 2012; Rodriguez et al., 2018). The cultures were maintained at 37°C on a heat-controlled stage, and the signals were sampled at 20 kHz with an MEA1060-Inv-BC amplifier (Multi Channel Systems).

### Signal Processing of In Vitro MEA Data

The signals were processed as described in Rodriguez et al. (2018). Briefly, the raw data were imported into MATLAB (MathWorks, Inc.) using MEA-Tools and filtered through a fourth-order Butterworth filter (20–2,000 Hz) and a notch filter to remove 60 Hz electric hum. Electrodes with irregular activity or excessive noise were excluded. The MATLAB routines used for signal processing and data analysis are described in (Kutzing et al., 2011; Rodriguez et al., 2018).

Spikes were detected using an adaptive threshold and were defined as a signal with voltage exceeding 4.5 standard deviations times the background noise for a 10-sec window for each recording channel. Spikes were counted once when detected at their maximum absolute value. ISIs were included if the ISI was longer than 2 msec to prevent duplicate counting of spikes. Spike rate refers to the number of spikes divided by the recording time (300 sec). Active electrodes were those with firing rate ≥ the 75th percentile of the distribution

For spike sorting, we applied Wave clus (Quiroga et al., 2004), which calculates the wavelet transform for each spike and uses a set of the obtained wavelet coefficients as input for a clustering algorithm based on k-nearest neighbor interactions (Blatt et al., 1996). As we have done previously (Rodriguez et al., 2018), we used wavelet transform (Quiroga et al., 2004; Rey et al., 2015) and collected a spike time stamp and waveform cutout spanning 1 msec before and 2.2 msec after the spike absolute maxima. The cutouts were sorted with the Wave clus algorithm with minor manual tuning (Quiroga et al., 2004).

Burstlets were defined as closely occurring spikes on an individual electrode according to the method used in Wagenaar et al. (2005). Spikes were determined to be part of a burstlet if there were at least four spikes with ISIs of the lesser of 100 msec or four times the firing rate of that electrode. Once the core group of spikes within a burstlet was identified, peripheral spikes (spikes at either the beginning or the end of the burstlet) were included if they had ISIs of the lesser of 200 msec or three times the firing rate of that electrode.

### Functional Connectivity Analysis

For our functional connectivity networks, we defined the nodes as the electrodes (for the in vitro case) or the neurons (for the in silico case) and the edges as the maximal cross-correlation within a predefined latency period between the two nodes. We computed the pairwise cross-correlation between spike trains in MATLAB (MathWorks) using xcorr. For both the in vitro MEA recordings and the in silico neuron network model, we binned the spikes into 1-msec time windows and enforced a 20-msec maximum lag time to only include short latency interactions. We then selected the maximum normalized cross-correlation within the defined latency period.

### Network Analysis

To investigate information exchange and community structure in the networks, we used the Brain Connectivity Toolbox (Rubinov & Sporns, 2010) to compute global efficiency, local efficiency, number of communities, and community statistic *Q* for both in silico and in vitro networks. Global efficiency is defined as the average inverse shortest path length and reflects how easily information is exchanged in the network. It measures network integration. To identify communities in our networks, we used the Louvain algorithm, which subdivides the network into nonoverlapping groups of nodes by maximizing within-community connectivity and minimizing between-community connectivity. The community statistic *Q* (modularity) quantifies how well the network can be subdivided into clearly defined communities compared with a random network. A higher *Q* signifies dense within-community connectivity relative to between-community connectivity. It measures network segregation. We also calculated local efficiency for in vitro networks since the nodes are defined as electrodes, not single neurons, to understand how efficiency is changing at the level of small groups of neurons (since each electrode on an MEA records from a few neurons). Local efficiency measures how efficient information flow is between neighbors when a node is removed. We calculated the weighted local efficiency based on Wang et al. (2017) and excluded baseline local efficiency values less than 0.001 to prevent aberrant normalization.

### Computational Neuron Network Model

This model was developed in Python for use with the Brian2 neural simulator (Stimberg et al., 2019). Based on Masquelier and Deco (2013), the network consists of 500 neurons (400 excitatory neurons, 100 inhibitory neurons). These neurons were connected in a probabilistic manner based on the type of connection: excitatory-excitatory connections had a 0.1 probability of connection, excitatory-inhibitory connections had a 0.05 probability, inhibitory-excitatory connections had a 0.2 probability, and inhibitory-inhibitory connections had a 0.2 probability. The neurons function as conductance-based, leaky integrate-and-fire neurons, and their membrane potential *V* follow the Langevin equation:

$$C_{m}\frac{dV}{dt}=-g_{m}(V-V_{L})-I_{syn}+I_{AHP}+I_{pre},$$

where *C*_*m*_ is the capacitance, *g*_*m*_ = 1/*R*_*m*_ is the membrane leak conductance, and *V*_*L*_ is the resting potential. The synaptic current (*I*_*syn*_) is defined as the sum of the glutamatergic AMPA and NMDA excitatory currents, while the after-hyperpolarization (*I*_*AHP*_) accounts for slow calcium dynamics and fatigue, and the final term (*I*_*pre*_) represents presynaptic noise currents, modeled as a Gaussian white noise function.

In our model, all synapses are modulated by short-term plasticity (Masquelier & Deco, 2013) and excitatory-excitatory connections were modulated by spike time–dependent plasticity (Bi & Poo, 2001).

For our simulations, neurons were seeded with a Gaussian distribution of initial external current inputs, and the network was allowed to stabilize for 10 sec before running for 300 sec. When observing the preliminary simulation results, we noticed brief, aperiodic bursting behavior at the beginning of our simulations uncharacteristic of the periodic bursting that appeared in the majority of the simulation. To determine an appropriate stabilization period to omit this aberrant behavior, we evaluated stabilization periods of 0, 2, 5, 10, 20, and 30 sec by calculating the effect of different settling periods on the interburst interval. We defined the interburst interval (IBI) as the time between burst events and defined burst events as at least 50 spikes occurring within a 50-msec period similar to Masquelier and Deco (2013). We found no significant difference between IBIs across stabilization periods. Out of an abundance of caution and visual inspection, we decided to deem the first 10 sec as the stabilization period. We used Euler forward integration with a time step of 0.1 msec to solve the differential equations. Each simulation was run five times, and the networks were analyzed in the same manner as the in vitro MEA recordings in MATLAB, examining the spike rate, ISI, FF, and coefficient of variation of the ISI as described in Rodriguez et al. (2018), as well as global efficiency, number of communities, and community statistic *Q* as described in Methods: Network Analysis.

### Analysis of In Silico Network Activity

For our simulations, we defined burst rate in the simulations as the number of bursts (50 spikes occurring within a 50 msec period) over the full simulation time of 300 sec. In comparison, the burst rate for in vitro MEA recordings was defined as four events with a maximum ISI between two consecutive spikes of 100 msec; additional spikes within 200 msec of the burst core or one-third times the electrode firing rate were included as part of the burst train (adapted from Chiappalone et al., 2007). These two different methods were applied because of the disparate ways in which bursts manifested in their respective networks. Note that although MEAs record from neural ensembles rather than single neurons as we modeled here, we do not expect qualitative differences in the aforementioned output metrics.

The code used to generate our neuron network model can be found at the Meaney Lab website (https://www.seas.upenn.edu/∼molneuro/).

### Cypin Overexpression in the Computational Model

To mimic the effect of cypin overexpression as described in the in vitro data in this report, we modulated (1) the AMPA receptor conductance to reflect the effect of cypin overexpression on AMPA-mediated signal transmission; (2) the connection density to reflect cypin-promoted changes to PSD-95 density at postsynaptic sites, and from earlier reports, cypin-promoted changes in spine density (Rodriguez et al., 2018); and (3) the amount of external current input to reflect cypin-promoted changes in mEPSC frequency. We optimized our cypin overexpression parameters to match the relative changes reported in the in vitro data for spike rate, ISI, burst rate, FF, and coefficient of variation. Additionally, we generated functional connectivity matrices as above (see Methods: Functional Connectivity Analysis) to investigate the network parameters global efficiency, number of communities, and community statistic *Q* and optimize our cypin overexpression parameters further.

### Training and Testing Response to Conditioned Stimulus

To understand how cypin overexpression affects information transfer in the network, we evaluated network response to conditioned stimulus as in (Gabrieli et al., 2021). Following stabilization of the network activity, we computed network activity for 300 sec and defined this as the control period. Next, we stimulated 1% of the excitatory neurons (input neurons) by doubling the external current they received for an additional 300 sec, labeling this phase as the training period. During the training period, we allowed the neurons in the network to ingrain learned responses to the stimulus. The testing period consisted of an additional 300 sec of activity following the training period. In the testing period, we continued to simulate the input neurons while we measured the firing rate of the output (noninput excitatory) neurons. We reported the signal fidelity as the ratio of the firing rate of the output neurons to the firing rate of the input neurons. We also conducted network analysis on the functional connectivity matrices for the pretraining control period and the posttraining period, and computed the change in the global efficiency, number of communities, and community statistic *Q* after training.

### Statistics

Data are presented as mean ± standard error of the mean. Statistical analyses were performed using Prism 7.0 (GraphPad, La Jolla, CA) and MATLAB; *p* < 0.05 is considered statistically significant. Comparisons of in vitro MEA data were performed using repeated-measures ANOVA followed by Tukey’s multiple comparisons test. Comparisons between simulated cypin overexpression networks and in vitro cypin overexpression recordings were performed with Welch’s correction for unequal variance.

## AUTHOR CONTRIBUTIONS

Ana R. Rodriguez: Data curation; Formal analysis; Investigation; Methodology; Writing – original draft. Erin D. Anderson: Formal analysis; Investigation; Methodology; Writing – review and editing. Kate M. O’Neill: Data curation; Formal analysis; Investigation; Methodology; Writing – review and editing. Przemyslaw P. McEwan: Data curation; Formal analysis; Investigation; Methodology; Writing – review and editing. Nicholas F. Vigilante: Investigation; Methodology. Munjin Kwon: Data curation; Formal analysis; Methodology. Barbara F. Akum: Formal analysis; Investigation; Methodology. Tamara M. Stawicki: Formal analysis; Investigation; Methodology. David F. Meaney: Conceptualization; Formal analysis; Project administration; Supervision; Writing – original draft; Writing – review and editing. Bonnie L. Firestein: Conceptualization; Formal analysis; Funding acquisition; Project administration; Supervision; Writing – original draft; Writing – review and editing.

## FUNDING INFORMATION

Bonnie L. Firestein, Division of Integrative Organismal Systems (http://dx.doi.org/10.13039/100000154), Award ID: IOS-1353724. Bonnie L. Firestein, New Jersey Commission on Brain Injury Research (http://dx.doi.org/10.13039/100008474), Award ID: CBIR14IRG019. Ana R. Rodriguez, National Institute of General Medical Sciences (http://dx.doi.org/10.13039/100000057), Award ID: T32 GM008339-20. Kate M. O’Neill, National Institute of General Medical Sciences (http://dx.doi.org/10.13039/100000057), Award ID: T32 GM008339-20. Przemyslaw P. McEwan, National Institute of General Medical Sciences (http://dx.doi.org/10.13039/100000057), Award ID: T32 GM008339-20. David F. Meaney, New Jersey Commission on Brain Injury Research (http://dx.doi.org/10.13039/100008474), Award ID: CBIR14IR G019. Kate M. O’Neill, New Jersey Commission on Brain Injury Research (http://dx.doi.org/10.13039/100008474), Award ID: CBIR13FEL002. Przemyslaw P. McEwan, New Jersey Commission on Brain Injury Research (http://dx.doi.org/10.13039/100008474), Award ID: CBIR16FEL013. Kate M. O’Neill, U.S. Department of Education (http://dx.doi.org/10.13039/100000138), Award ID: P200A150131. Tamara M. Stawicki, Rutgers, The State University of New Jersey (http://dx.doi.org/10.13039/100011132), Award ID: Rutgers University School of Arts and Sciences Honors Program Research Fellowship. Bonnie L. Firestein, New Jersey Commission on Brain Injury Research (http://dx.doi.org/10.13039/100008474), Award ID: CBIR20IRG003. David F. Meaney, New Jersey Commission on Brain Injury Research (http://dx.doi.org/10.13039/100008474), Award ID: CBIR20IRG003.

## TECHNICAL TERMS

Synaptogenesis: The formation of synapses in neurons, usually during early brain development.
Postsynaptic density (PSD): Postsynaptic neuron membrane region containing dense meshwork of cytoskeletal and signaling proteins, specialized for transmitting information received at the synapse.
Synaptosomes: Synaptic terminals that have been isolated through biochemical techniques.
Miniature excitatory postsynaptic currents (mEPSCs): Electrical activity in the postsynaptic neuron that results from excitatory neurotransmitter release from the presynaptic neuron.
Microelectrode array (MEA): A device that contains many micron-sized electrodes that record local field potentials from cultured cells (also called a multielectrode array).
Fano factor: Describes spike count variability; the ratio of the variance to the mean of spike counts within a specific time interval.
Coefficient of variation: Describes spike timing variability; the ratio of the standard deviation to the mean of the interspike interval distribution.
Efficiency: How easily information traverses the network; global when considered across the entire network, local when considered amongst a node’s direct neighbors.
Conductance: How easily charge (ions) crosses the membrane; an increase in conductance corresponds to an increase in current through the membrane.
Community: A group within the network identified by maximizing the within-community connectivity while minimizing between-community connectivity.
Excitatory neuron: Presynaptic neuron that releases excitatory neurotransmitters that bind receptors (e.g., NMDAR, AMPAR) on the postsynaptic neuron, increasing axon potential likelihood.

## References

[bib1] Akum, B. F., Chen, M., Gunderson, S. I., Riefler, G. M., Scerri-Hansen, M. M., & Firestein, B. L. (2004). Cypin regulates dendrite patterning in hippocampal neurons by promoting microtubule assembly. Nature Neuroscience, 7(2), 145–152. **DOI:** https://doi.org/10.1038/nn1179, **PMID:** 14730308 14730308 10.1038/nn1179

[bib2] Azarfar, A., Calcini, N., Huang, C., Zeldenrust, F., & Celikel, T. (2018). Neural coding: A single neuron’s perspective. Neuroscience & Biobehavioral Reviews, 94, 238–247. **DOI:** https://doi.org/10.1016/j.neubiorev.2018.09.007, **PMID:** 30227142 30227142 10.1016/j.neubiorev.2018.09.007

[bib3] Bai, F., & Witzmann, F. A. (2007). Synaptosome proteomics. Subcellular Biochemistry, 43, 77–98. **DOI:** https://doi.org/10.1007/978-1-4020-5943-8_6, **PMID:** 17953392, **PMCID:** PMC2853956 17953392 10.1007/978-1-4020-5943-8_6 PMC2853956

[bib4] Beique, J. C., Lin, D. T., Kang, M. G., Aizawa, H., Takamiya, K., & Huganir, R. L. (2006). Synapse-specific regulation of AMPA receptor function by PSD-95. Proceedings of the National Academy of Sciences of the United States of America, 103(51), 19535–19540. **DOI:** https://doi.org/10.1073/pnas.0608492103, **PMID:** 17148601, **PMCID:** PMC1748260 17148601 10.1073/pnas.0608492103 PMC1748260

[bib5] Betzel, R. F., Satterthwaite, T. D., Gold, J. I., & Bassett, D. S. (2017). Positive affect, surprise, and fatigue are correlates of network flexibility. Scientific Reports, 7(1), 520. **DOI:** https://doi.org/10.1038/s41598-017-00425-z, **PMID:** 28364117, **PMCID:** PMC5428446 28364117 10.1038/s41598-017-00425-z PMC5428446

[bib6] Bi, G., & Poo, M. (2001). Synaptic modification by correlated activity: Hebb’s postulate revisited. Annual Review of Neuroscience, 24, 139–166. **DOI:** https://doi.org/10.1146/annurev.neuro.24.1.139, **PMID:** 11283308 10.1146/annurev.neuro.24.1.139 11283308

[bib7] Bingol, B., & Schuman, E. M. (2004). A proteasome-sensitive connection between PSD-95 and GluR1 endocytosis. Neuropharmacology, 47(5), 755–763. **DOI:** https://doi.org/10.1016/j.neuropharm.2004.07.028, **PMID:** 15458847 15458847 10.1016/j.neuropharm.2004.07.028

[bib8] Blatt, M., Wiseman, S., & Domany, E. (1996). Superparamagnetic clustering of data. Physical Review Letters, 76(18), 3251–3254. **DOI:** https://doi.org/10.1103/PhysRevLett.76.3251, **PMID:** 10060920 10060920 10.1103/PhysRevLett.76.3251

[bib9] Bredt, D. S., & Nicoll, R. A. (2003). AMPA receptor trafficking at excitatory synapses. Neuron, 40(2), 361–379. 10.1016/S0896-6273(03)00640-8 14556714

[bib10] Brenman, J. E., Chao, D. S., Gee, S. H., McGee, A. W., Craven, S. E., Santillano, D. R., Wu, Z., Huang, F., Xia, H., Peters, M. F., Froehner, S. C., & Bredt, D. S. (1996). Interaction of nitric oxide synthase with the postsynaptic density protein PSD-95 and alpha1-syntrophin mediated by PDZ domains. Cell, 84(5), 757–767. 10.1016/S0092-8674(00)81053-3 8625413

[bib11] Bressler, S. L., & Kelso, J. A. (2001). Cortical coordination dynamics and cognition. Trends in Cognitive Sciences, 5(1), 26–36. 10.1016/S1364-6613(00)01564-3 11164733

[bib12] Capone, C., Gigante, G., & Del Giudice, P. (2018). Spontaneous activity emerging from an inferred network model captures complex spatio-temporal dynamics of spike data. Scientific Reports, 8(1), 17056. **DOI:** https://doi.org/10.1038/s41598-018-35433-0, **PMID:** 30451957, **PMCID:** PMC6242821 30451957 10.1038/s41598-018-35433-0 PMC6242821

[bib13] Charych, E. I., Akum, B. F., Goldberg, J. S., Jornsten, R. J., Rongo, C., Zheng, J. Q., & Firestein, B. L. (2006). Activity-independent regulation of dendrite patterning by postsynaptic density protein PSD-95. The Journal of Neuroscience, 26(40), 10164–10176. **DOI:** https://doi.org/10.1523/JNEUROSCI.2379-06.2006, **PMID:** 17021172, **PMCID:** PMC6674632 17021172 10.1523/JNEUROSCI.2379-06.2006 PMC6674632

[bib14] Chen, H., & Firestein, B. L. (2007). RhoA regulates dendrite branching in hippocampal neurons by decreasing cypin protein levels. The Journal of Neuroscience, 27(31), 8378–8386. **DOI:** https://doi.org/10.1523/JNEUROSCI.0872-07.2007, **PMID:** 17670984, **PMCID:** PMC6673065 17670984 10.1523/JNEUROSCI.0872-07.2007 PMC6673065

[bib15] Chen, M., Lucas, K. G., Akum, B. F., Balasingam, G., Stawicki, T. M., Provost, J. M., Riefler, G. M., Jornsten, R. J., & Firestein, B. L. (2005). A novel role for Snapin in dendrite patterning: Interaction with cypin. Molecular Biology of the Cell, 16, 5103–5114. **DOI:** https://doi.org/10.1091/mbc.e05-02-0165, **PMID:** 16120643, **PMCID:** PMC1266411 16120643 10.1091/mbc.E05-02-0165 PMC1266411

[bib16] Chiappalone, M., Vato, A., Berdondini, L., Koudelka-Hep, M., & Martinoia, S. (2007). Network dynamics and synchronous activity in cultured cortical neurons. International Journal of Neural Systems, 17(2), 87–103. **DOI:** https://doi.org/10.1142/S0129065707000968, **PMID:** 17565505 17565505 10.1142/S0129065707000968

[bib17] Cho, K. O., Hunt, C. A., & Kennedy, M. B. (1992). The rat brain postsynaptic density fraction contains a homolog of the Drosophila discs-large tumor suppressor protein. Neuron, 9(5), 929–942. 10.1016/0896-6273(92)90245-9 1419001

[bib18] Cohen, N. A., Brenman, J. E., Snyder, S. H., & Bredt, D. S. (1996). Binding of the inward rectifier K+ channel Kir 2.3 to PSD-95 is regulated by protein kinase A phosphorylation. Neuron, 17(4), 759–767. 10.1016/S0896-6273(00)80207-X 8893032

[bib19] Colledge, M., Snyder, E. M., Crozier, R. A., Soderling, J. A., Jin, Y., Langeberg, L. K., Lu, H., Bear, M. F., & Scott, J. D. (2003). Ubiquitination regulates PSD-95 degradation and AMPA receptor surface expression. Neuron, 40(3), 595–607. 10.1016/S0896-6273(03)00687-1 14642282 PMC3963808

[bib20] Ehlers, M. D. (2003). Activity level controls postsynaptic composition and signaling via the ubiquitin-proteasome system. Nature Neuroscience, 6(3), 231–242. **DOI:** https://doi.org/10.1038/nn1013, **PMID:** 12577062 12577062 10.1038/nn1013

[bib21] El-Husseini, A. E., Schnell, E., Chetkovich, D. M., Nicoll, R. A., & Bredt, D. S. (2000). PSD-95 involvement in maturation of excitatory synapses. Science, 290(5495), 1364–1368. **DOI:** https://doi.org/10.1126/science.290.5495.1364, **PMID:** 11082065 11082065

[bib22] Fardet, T., Ballandras, M., Bottani, S., Metens, S., & Monceau, P. (2018). Understanding the generation of network bursts by adaptive oscillatory neurons. Frontiers in Neuroscience, 12, 41. **DOI:** https://doi.org/10.3389/fnins.2018.00041, **PMID:** 29467607, **PMCID:** PMC5808224 29467607 10.3389/fnins.2018.00041 PMC5808224

[bib23] Firestein, B. L., Brenman, J. E., Aoki, C., Sanchez-Perez, A. M., El-Husseini, A. E., & Bredt, D. S. (1999). Cypin: A cytosolic regulator of PSD-95 postsynaptic targeting. Neuron, 24(3), 659–672. 10.1016/S0896-6273(00)81120-4 10595517

[bib24] Fong, M. F., Newman, J. P., Potter, S. M., & Wenner, P. (2015). Upward synaptic scaling is dependent on neurotransmission rather than spiking. Nature Communications, 6, 6339. **DOI:** https://doi.org/10.1038/ncomms7339, **PMID:** 25751516, **PMCID:** PMC4355957 10.1038/ncomms7339 PMC4355957 25751516

[bib25] Gabrieli, D., Schumm, S. N., Vigilante, N. F., & Meaney, D. F. (2021). NMDA receptor alterations after mild traumatic brain injury induce deficits in memory acquisition and recall. Neural Computation, 33(1), 67–95. **DOI:** https://doi.org/10.1162/neco_a_01343, **PMID:** 33253030, **PMCID:** PMC7856344 33253030 10.1162/neco_a_01343 PMC7856344

[bib26] Gallistel, C. R. (2017). The coding question. Trends in Cognitive Sciences, 21(7), 498–508. **DOI:** https://doi.org/10.1016/j.tics.2017.04.012, **PMID:** 28522379 28522379 10.1016/j.tics.2017.04.012

[bib27] Haber, S. N., & Calzavara, R. (2009). The cortico-basal ganglia integrative network: the role of the thalamus. Brain Research Bulletin, 78(2–3), 69–74. **DOI:** https://doi.org/10.1016/j.brainresbull.2008.09.013, **PMID:** 18950692, **PMCID:** PMC4459637 18950692 10.1016/j.brainresbull.2008.09.013 PMC4459637

[bib28] Harmony, T. (2013). The functional significance of delta oscillations in cognitive processing. Frontiers in Integrative Neuroscience, 7, 83. **DOI:** https://doi.org/10.3389/fnint.2013.00083, **PMID:** 24367301, **PMCID:** PMC3851789 24367301 10.3389/fnint.2013.00083 PMC3851789

[bib29] Hausser, M. (2001). Synaptic function: Dendritic democracy. Current Biology, 11(1), R10–R12. **DOI:** https://doi.org/10.1016/S0960-9822(00)00034-8, **PMID:** 11166188 11166188 10.1016/s0960-9822(00)00034-8

[bib30] Hernandez, K., Swiatkowski, P., Patel, M. V., Liang, C., Dudzinski, N. R., Brzustowicz, L. M., & Firestein, B. L. (2016). Overexpression of isoforms of nitric oxide synthase 1 adaptor protein, encoded by a risk gene for schizophrenia, alters actin dynamics and synaptic function. Frontiers in Cellular Neuroscience, 10, 6. **DOI:** https://doi.org/10.3389/fncel.2016.00006, **PMID:** 26869880, **PMCID:** PMC4735351 26869880 10.3389/fncel.2016.00006 PMC4735351

[bib31] Hwang, K., Bertolero, M. A., Liu, W. B., & D’Esposito, M. (2017). The Human thalamus is an integrative hub for functional brain networks. The Journal of Neuroscience, 37(23), 5594–5607. **DOI:** https://doi.org/10.1523/JNEUROSCI.0067-17.2017, **PMID:** 28450543, **PMCID:** PMC5469300 28450543 10.1523/JNEUROSCI.0067-17.2017 PMC5469300

[bib32] Ilardi, J. M., Mochida, S., & Sheng, Z. H. (1999). Snapin: A SNARE-associated protein implicated in synaptic transmission. Nature Neuroscience, 2(2), 119–124. **DOI:** https://doi.org/10.1038/5673, **PMID:** 10195194 10195194 10.1038/5673

[bib33] Katz, Y., Menon, V., Nicholson, D. A., Geinisman, Y., Kath, W. L., & Spruston, N. (2009). Synapse distribution suggests a two-stage model of dendritic integration in CA1 pyramidal neurons. Neuron, 63(2), 171–177. **DOI:** https://doi.org/10.1016/j.neuron.2009.06.023, **PMID:** 19640476, **PMCID:** PMC2921807 19640476 10.1016/j.neuron.2009.06.023 PMC2921807

[bib34] Keith, D., & El-Husseini, A. (2008). Excitation control: balancing PSD-95 function at the synapse. Frontiers in Molecular Neuroscience, 1, 4. **DOI:** https://doi.org/10.3389/neuro.02.004.2008, **PMID:** 18946537, **PMCID:** PMC2526002 18946537 10.3389/neuro.02.004.2008 PMC2526002

[bib35] Kim, E., Niethammer, M., Rothschild, A., Jan, Y. N., & Sheng, M. (1995). Clustering of Shaker-type K+ channels by interaction with a family of membrane-associated guanylate kinases. Nature, 378(6552), 85–88. **DOI:** https://doi.org/10.1038/378085a0, **PMID:** 7477295 7477295 10.1038/378085a0

[bib36] Kim, E., & Sheng, M. (2004). PDZ domain proteins of synapses. Nature Reviews Neuroscience, 5(10), 771–781. **DOI:** https://doi.org/10.1038/nrn1517, **PMID:** 15378037 15378037 10.1038/nrn1517

[bib37] Kim, J. H., Liao, D., Lau, L. F., & Huganir, R. L. (1998). SynGAP: a synaptic rasGAP that associates with the PSD-95/SAP90 protein family. Neuron, 20(4), 683–691. 10.1016/S0896-6273(00)81008-9 9581761

[bib38] Kistner, U., Wenzel, B. M., Veh, R. W., Cases-Langhoff, C., Garner, A. M., Appeltauer, U., Voss, B., Gundelfinger, E. D., & Garner, C. C. (1993). SAP90, a rat presynaptic protein related to the product of the Drosophila tumor suppressor gene dlg-A. Journal of Biological Chemistry, 268(7), 4580–4583. **DOI:** https://doi.org/10.1016/S0021-9258(18)53433-5, **PMID:** 7680343 7680343

[bib39] Kornau, H. C., Schenker, L. T., Kennedy, M. B., & Seeburg, P. H. (1995). Domain interaction between NMDA receptor subunits and the postsynaptic density protein PSD-95. Science, 269(5231), 1737–1740. **DOI:** https://doi.org/10.1126/science.7569905, **PMID:** 7569905 7569905 10.1126/science.7569905

[bib40] Kulkarni, V. A., & Firestein, B. L. (2012). The dendritic tree and brain disorders. Molecular and Cellular Neuroscience, 50(1), 10–20. **DOI:** https://doi.org/10.1016/j.mcn.2012.03.005, **PMID:** 22465229 22465229 10.1016/j.mcn.2012.03.005

[bib41] Kutzing, M. K., Luo, V., & Firestein, B. L. (2011). Measurement of synchronous activity by microelectrode arrays uncovers differential effects of sublethal and lethal glutamate concentrations on cortical neurons. Annals of Biomedical Engineering, 39(8), 2252–2262. **DOI:** https://doi.org/10.1007/s10439-011-0319-0, **PMID:** 21544673 21544673 10.1007/s10439-011-0319-0

[bib42] Kutzing, M. K., Luo, V., & Firestein, B. L. (2012). Protection from glutamate-induced excitotoxicity by memantine. Annals of Biomedical Engineering, 40(5), 1170–1181. **DOI:** https://doi.org/10.1007/s10439-011-0494-z, **PMID:** 22203191, **PMCID:** PMC4217523 22203191 10.1007/s10439-011-0494-z PMC4217523

[bib43] Marrs, G. S., Green, S. H., & Dailey, M. E. (2001). Rapid formation and remodeling of postsynaptic densities in developing dendrites. Nature Neuroscience, 4(10), 1006–1013. **DOI:** https://doi.org/10.1038/nn717, **PMID:** 11574832 11574832 10.1038/nn717

[bib44] Masquelier, T., & Deco, G. (2013). Network bursting dynamics in excitatory cortical neuron cultures results from the combination of different adaptive mechanisms. PLoS One, 8(10), e75824. **DOI:** https://doi.org/10.1371/journal.pone.0075824, **PMID:** 24146781, **PMCID:** PMC3795681 24146781 10.1371/journal.pone.0075824 PMC3795681

[bib45] Matus, A. (2000). Actin-based plasticity in dendritic spines. Science, 290(5492), 754–758. **DOI:** https://doi.org/10.1126/science.290.5492.754, **PMID:** 11052932 11052932 10.1126/science.290.5492.754

[bib46] Mayford, M. (2014). The search for a hippocampal engram. Philosophical Transactions of the Royal Society B: Biological Sciences, 369(1633), 20130161. **DOI:** https://doi.org/10.1098/rstb.2013.0161, **PMID:** 24298162, **PMCID:** PMC3843892 10.1098/rstb.2013.0161 PMC3843892 24298162

[bib47] McCormick, D. A., & Contreras, D. (2001). On the cellular and network bases of epileptic seizures. Annual Review of Physiology, 63, 815–846. **DOI:** https://doi.org/10.1146/annurev.physiol.63.1.815, **PMID:** 11181977 10.1146/annurev.physiol.63.1.815 11181977

[bib48] Nyhus, E., & Curran, T. (2010). Functional role of gamma and theta oscillations in episodic memory. Neuroscience & Biobehavioral Reviews, 34(7), 1023–1035. **DOI:** https://doi.org/10.1016/j.neubiorev.2009.12.014, **PMID:** 20060015, **PMCID:** PMC2856712 20060015 10.1016/j.neubiorev.2009.12.014 PMC2856712

[bib49] O’Neill, K. M., Donohue, K. E., Omelchenko, A., & Firestein, B. L. (2018). The 3′UTRs of brain-derived neurotrophic factor transcripts differentially regulate the dendritic arbor. Frontiers in Cellular Neuroscience, 12, 60. **DOI:** https://doi.org/10.3389/fncel.2018.00060, **PMID:** 29563866, **PMCID:** PMC5845904 29563866 10.3389/fncel.2018.00060 PMC5845904

[bib50] O’Neill, K. M., Kwon, M., Donohue, K. E., & Firestein, B. L. (2017). Distinct effects on the dendritic arbor occur by microbead versus bath administration of brain-derived neurotrophic factor. Cellular and Molecular Life Sciences, 74(23), 4369–4385. **DOI:** https://doi.org/10.1007/s00018-017-2589-7, **PMID:** 28698933, **PMCID:** PMC5819737 28698933 10.1007/s00018-017-2589-7 PMC5819737

[bib51] Okabe, S., Kim, H. D., Miwa, A., Kuriu, T., & Okado, H. (1999). Continual remodeling of postsynaptic density and its regulation by synaptic activity. Nature Neuroscience, 2(9), 804–811. **DOI:** https://doi.org/10.1038/12175, **PMID:** 10461219 10461219 10.1038/12175

[bib52] Pak, D. T., Yang, S., Rudolph-Correia, S., Kim, E., & Sheng, M. (2001). Regulation of dendritic spine morphology by SPAR, a PSD-95-associated rapGAP. Neuron, 31(2), 289–303. 10.1016/S0896-6273(01)00355-5 11502259

[bib53] Pan, P. Y., Tian, J. H., & Sheng, Z. H. (2009). Snapin facilitates the synchronization of synaptic vesicle fusion. Neuron, 61(3), 412–424. **DOI:** https://doi.org/10.1016/j.neuron.2008.12.029, **PMID:** 19217378, **PMCID:** PMC2656773 19217378 10.1016/j.neuron.2008.12.029 PMC2656773

[bib54] Patel, M. V., Swiatkowski, P., Kwon, M., Rodriguez, A. R., Campagno, K., & Firestein, B. L. (2018). A novel short isoform of cytosolic PSD-95 interactor (cypin) regulates neuronal development. Molecular Neurobiology, 55(8), 6269–6281. **DOI:** https://doi.org/10.1007/s12035-017-0849-z, **PMID:** 29294243, **PMCID:** PMC6028325 29294243 10.1007/s12035-017-0849-z PMC6028325

[bib55] Ponulak, F., & Kasinski, A. (2011). Introduction to spiking neural networks: Information processing, learning and applications. Acta Neurobiologiae Experimentalis, 71(4), 409–433. 22237491 10.55782/ane-2011-1862

[bib56] Prange, O., & Murphy, T. H. (2001). Modular transport of postsynaptic density-95 clusters and association with stable spine precursors during early development of cortical neurons. The Journal of Neuroscience, 21(23), 9325–9333. **DOI:** https://doi.org/10.1523/JNEUROSCI.21-23-09325.2001, **PMID:** 11717366, **PMCID:** PMC6763916 11717366 10.1523/JNEUROSCI.21-23-09325.2001 PMC6763916

[bib57] Quiroga, R. Q., Nadasdy, Z., & Ben-Shaul, Y. (2004). Unsupervised spike detection and sorting with wavelets and superparamagnetic clustering. Neural Computation, 16(8), 1661–1687. **DOI:** https://doi.org/10.1162/089976604774201631, **PMID:** 15228749 15228749 10.1162/089976604774201631

[bib58] Rey, H. G., Pedreira, C., & Quian Quiroga, R. (2015). Past, present and future of spike sorting techniques. Brain Research Bulletin, 119(Pt B), 106–117. **DOI:** https://doi.org/10.1016/j.brainresbull.2015.04.007, **PMID:** 25931392, **PMCID:** PMC4674014 25931392 10.1016/j.brainresbull.2015.04.007 PMC4674014

[bib59] Rodriguez, A. R., O’Neill, K. M., Swiatkowski, P., Patel, M. V., & Firestein, B. L. (2018). Overexpression of cypin alters dendrite morphology, single neuron activity, and network properties via distinct mechanisms. Journal of Neural Engineering, 15(1), 016020. **DOI:** https://doi.org/10.1088/1741-2552/aa976a, **PMID:** 29091046, **PMCID:** PMC5797707 29091046 10.1088/1741-2552/aa976a PMC5797707

[bib60] Rozeske, R. R., & Herry, C. (2018). Neuronal coding mechanisms mediating fear behavior. Current Opinion in Neurobiology, 52, 60–64. **DOI:** https://doi.org/10.1016/j.conb.2018.04.017, **PMID:** 29705550 29705550 10.1016/j.conb.2018.04.017

[bib61] Rubinov, M., & Sporns, O. (2010). Complex network measures of brain connectivity: uses and interpretations. NeuroImage, 52(3), 1059–1069. **DOI:** https://doi.org/10.1016/j.neuroimage.2009.10.003, **PMID:** 19819337 19819337 10.1016/j.neuroimage.2009.10.003

[bib62] Scott, K., & Zuker, C. (1997). Lights out: deactivation of the phototransduction cascade. Trends in Biochemical Sciences, 22(9), 350–354. 10.1016/S0968-0004(97)01100-6 9301336

[bib63] Scott, K., & Zuker, C. (1998). TRP, TRPL and trouble in photoreceptor cells. Current Opinion in Neurobiology, 8(3), 383–388. 10.1016/S0959-4388(98)80065-2 9687362

[bib64] Sheng, M., & Wyszynski, M. (1997). Ion channel targeting in neurons. Bioessays, 19(10), 847–853. **DOI:** https://doi.org/10.1002/bies.950191004, **PMID:** 9363678 9363678 10.1002/bies.950191004

[bib65] Sporns, O., & Betzel, R. F. (2016). Modular brain networks. Annual Review of Psychology, 67, 613–640. **DOI:** https://doi.org/10.1146/annurev-psych-122414-033634, **PMID:** 26393868, **PMCID:** PMC4782188 10.1146/annurev-psych-122414-033634 PMC4782188 26393868

[bib66] Steward, O., & Schuman, E. M. (2001). Protein synthesis at synaptic sites on dendrites. Annual Review of Neuroscience, 24, 299–325. **DOI:** https://doi.org/10.1146/annurev.neuro.24.1.299, **PMID:** 11283313 10.1146/annurev.neuro.24.1.299 11283313

[bib67] Stimberg, M., Brette, R., & Goodman, D. F. (2019). Brian 2, an intuitive and efficient neural simulator. eLife, 8, e47314. **DOI:** https://doi.org/10.7554/eLife.47314, **PMID:** 31429824, **PMCID:** PMC6786860 31429824 10.7554/eLife.47314 PMC6786860

[bib68] Swiatkowski, P., Sewell, E., Sweet, E. S., Dickson, S., Swanson, R. A., McEwan, S. A., Cuccolo, N., McDonnell, M. E., Patel, M. V., Varghese, N., Morrison, B., Reitz, A. B., Meaney, D. F., & Firestein, B. L. (2018). Cypin: A novel target for traumatic brain injury. Neurobiology of Disease, 119, 13–25. **DOI:** https://doi.org/10.1016/j.nbd.2018.07.019, **PMID:** 30031156, **PMCID:** PMC6214165 30031156 10.1016/j.nbd.2018.07.019 PMC6214165

[bib69] Tyagi, R. (2015). Neural networks of the mouse neocortex. Cell, 156(5), 1096–1111. **DOI:** https://doi.org/10.5214/ans.0972.7531.220409, **PMID:** 26525649, **PMCID:** PMC4627193 10.1016/j.cell.2014.02.023 PMC4169118 24581503

[bib70] Tyukin, I. Y., Iudin, D., Iudin, F., Tyukina, T., Kazantsev, V., Mukhina, I., & Gorban, A. N. (2019). Simple model of complex dynamics of activity patterns in developing networks of neuronal cultures. PLoS ONE, 14(6), e0218304. **DOI:** https://doi.org/10.1371/journal.pone.0218304, **PMID:** 31246978, **PMCID:** PMC6597067 31246978 10.1371/journal.pone.0218304 PMC6597067

[bib71] Vandenberghe, W., Nicoll, R. A., & Bredt, D. S. (2005). Stargazin is an AMPA receptor auxiliary subunit. Proceedings of the National Academy of Sciences of the United States of America, 102(2), 485–490. **DOI:** https://doi.org/10.1073/pnas.0408269102, **PMID:** 15630087, **PMCID:** PMC544314 15630087 10.1073/pnas.0408269102 PMC544314

[bib72] Wagenaar, D. A., DeMarse, T. B., & Potter, S. M. (2005). MeaBench: A toolset for multi-electrode data acquisition and on-line analysis. Proceedings of the 2nd International IEEE EMBS. 10.1109/CNE.2005.1419673

[bib73] Wang, Y., Ghumare, E., Vandenberghe, R., & Dupont, P. (2017). Comparison of different generalizations of clustering coefficient and local efficiency for weighted undirected graphs. Neural Computation, 29(2), 313–331. **DOI:** https://doi.org/10.1162/NECO_a_00914, **PMID:** 27870616 27870616 10.1162/NECO_a_00914

[bib74] Yudowski, G. A., Olsen, O., Adesnik, H., Marek, K. W., & Bredt, D. S. (2013). Acute inactivation of PSD-95 destabilizes AMPA receptors at hippocampal synapses. PLoS One, 8(1), e53965. **DOI:** https://doi.org/10.1371/journal.pone.0053965, **PMID:** 23342049, **PMCID:** PMC3546964 23342049 10.1371/journal.pone.0053965 PMC3546964

[bib75] Zingg, B., Hintiryan, H., Gou, L., Song, M. Y., Bay, M., Bienkowski, M. S., Foster, N. N., Yamashita, S., Bowman, I., Toga, A. W., & Dong, H. W. (2014). Neural networks of the mouse neocortex. Cell, 156(5), 1096–1111. **DOI:** https://doi.org/10.1016/j.cell.2014.02.023, **PMID:** 24581503, **PMCID:** PMC4169118 24581503 10.1016/j.cell.2014.02.023 PMC4169118

